# Enhanced preservation of the human intestinal microbiota by ridinilazole, a novel *Clostridium difficile*-targeting antibacterial, compared to vancomycin

**DOI:** 10.1371/journal.pone.0199810

**Published:** 2018-08-02

**Authors:** Cheleste M. Thorpe, Anne V. Kane, Justin Chang, Albert Tai, Richard J. Vickers, David R. Snydman

**Affiliations:** 1 Tufts Medical Center, Boston, MA, United States of America; 2 Tufts University School of Medicine, Boston, MA, United States of America; 3 Summit Therapeutics Plc, Abingdon, Oxfordshire, United Kingdom; University Hospital Llandough, UNITED KINGDOM

## Abstract

Ridinilazole, a novel targeted antibacterial being developed for the treatment of *C*. *difficile* infection (CDI) and prevention of recurrence, was shown in a recent Phase 2 study to be superior to vancomycin with regard to the primary efficacy measure, sustained clinical response (SCR), with the superiority being driven primarily by marked reductions in the rates of CDI recurrence within 30 days. Tolerability of ridinilazole was comparable to that of vancomycin. The current nested cohort study compared the effects of ridinilazole and vancomycin on fecal microbiota during and after treatment among participants in the Phase 2 study. Changes in the microbiota were assessed using qPCR and high-throughput sequencing on participants’ stools collected at multiple time-points (baseline [Day 1], Day 5, end-of-treatment [EOT; Day 10], Day 25, end-of-study [EOS; Day 40], and at CDI recurrence). qPCR analyses showed profound losses of *Bacteroides*, *C*. *coccoides*, *C*. *leptum*, and *Prevotella* groups at EOT with vancomycin treatment, while ridinilazole-treated participants had a modest decrease in *C*. *leptum* group levels at EOT, with levels recovering by Day 25. Vancomycin-treated participants had a significant increase in the Enterobacteriaceae group, with this increase persisting beyond EOT. At EOT, alpha diversity decreased with both antibiotics, though to a significantly lesser extent with ridinilazole (p <0.0001). Beta diversity analysis showed a significantly larger weighted Unifrac distance from baseline-to-EOT with vancomycin. Taxonomically, ridinilazole had a markedly narrower impact, with modest reductions in relative abundance in Firmicutes taxa. Microbiota composition returned to baseline sooner with ridinilazole than with vancomycin. Vancomycin treatment resulted in microbiome-wide changes, with significant reductions in relative abundances of Firmicutes, Bacteroidetes, Actinobacteria, and a profound increase in abundance of Proteobacteria. These findings demonstrate that ridinilazole is significantly less disruptive to microbiota than vancomycin, which may contribute to the reduced CDI recurrence observed in the Phase 2 study.

## Introduction

Over 450,000 cases of CDI occur in the US annually, with over 80,000 first recurrences and approximately 29,000 deaths [1]. The most common precipitant is antibiotic use. Antibiotics cause loss of colonization resistance with the potential establishment of a long-lasting, species-poor microbiota susceptible to pathogen invasion [2–5]. Oral vancomycin and metronidazole treatment are associated with high CDI recurrence rates, likely due to deleterious effects on resident colonic flora [6, 7]. Recurrences are costly in terms of both clinical burden and healthcare resource utilization [8, 9]. In one study, readmission was required in approximately one-third of recurrence cases [8].

Both microbiota biomass and composition at the intestinal-bacterial interface likely influence the *C*. *difficile* colonization niche [7]. Although colonization resistance has been associated with specific taxa [10–15], it is likely that different, yet diverse, microbiota community structures can confer protection. Consistent characteristics of communities susceptible to CDI are low diversity levels and diminished metabolic function [16] with loss of relative abundance of members of the Bacteroidetes and Firmicutes phyla and increases in that of Proteobacteria [17–21]. Fecal microbiota transplantation (FMT) normalizes these features and breaks the CDI recurrence cycle [22–26].

In aggregate, these data support a role for CDI agents with minimal effects on indigenous microbiota to reduce risk of recurrence. Ridinilazole (SMT19969) is a narrow-spectrum, non-absorbable, potent *C*. *difficile*-targeting antimicrobial [27]. In a recent Phase 2 randomized, controlled, double-blinded clinical trial comparing its efficacy to vancomycin, ridinilazole was associated with marked reduction in rates of recurrent disease (14.3% vs. 34.8%) [28]. The goal of the present study was to compare effects over time and treatment group on host fecal microbiota biomass and taxonomic composition in participants from the Phase 2 study. Fecal biomasses of specific microbial families (Eubacteria and 5 different microbiota groups; Bacteroides, *C*. *coccoides*, *C*. *leptum*, Enterobacteriaceae, and Prevotella) were quantified by qPCR. Relative abundances of resident taxa, microbiota diversity, and taxonomic composition were assessed by high-throughput sequencing.

## Materials and methods

### Participant enrollment and sample collection

**Fig 1:**
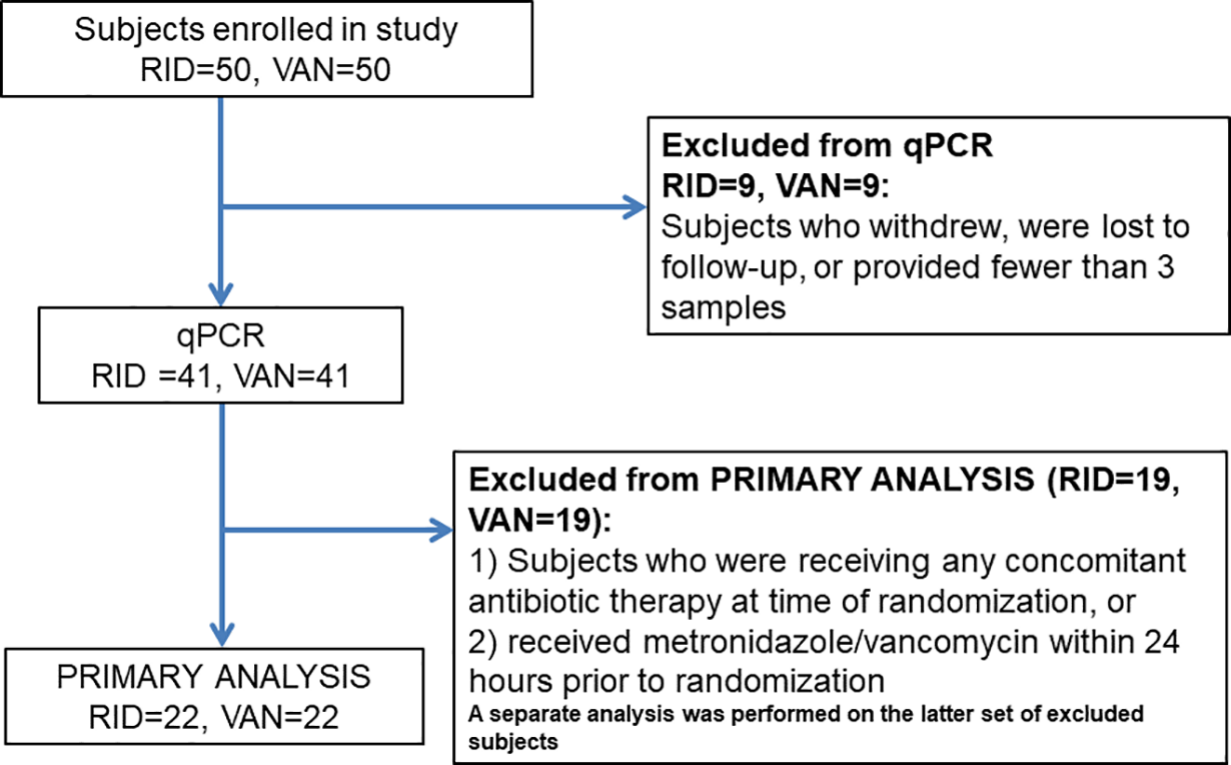
Consort diagram for participant inclusion in the nested cohort. Abbreviations: RDZ, ridinilazole; VAN, vancomycin.

One hundred patients were enrolled and randomized 1:1 to receive 10 days of either vancomycin or ridinilazole [28]. Institutional review boards at each center (see S1 Table for details) provided ethics approval. The study complied with ethical principles expressed in the Declaration of Helsinki and followed all principles of good clinical practice. Written informed consent was obtained from all participants. Stool samples were obtained at study entry (baseline, D1), day 5 (D5), day 10 (D10, EOT), day 25 (D25) and day 40 post-entry (D40, end-of-study, EOS), and if recurrence was suspected. In order to provide a healthy control benchmark for this study, control stool samples were obtained from age-, gender- and location-similar volunteers enrolled in a separate study [29], and handled similarly as the samples from CDI subjects in this study. Of 100 patients enrolled in the clinical trial, 18 did not provide at least 3 stool samples for analysis (Fig 1). Due to the fact that stool microbiota is highly individual-specific, the appropriate comparison is of the patient to themselves over the time of the study. Thus,18 subjects from whom a limited number of stools were collected were eliminated from this study. Of the remaining 82 participants, there were 41 per treatment arm. As any antibiotic therapy can alter microbiota, thus introducing confounding effects, some of the Phase 2 study subjects and samples were excluded from this nested cohort study in order to confine the analysis to studying the effect of ridinilazole or vancomycin only. Some subjects received concomitant non-CDI antibiotics during the study, so any sample obtained after initiation of treatment with non-CDI antibiotics was excluded. Similarly, samples were not included in the analysis if participants were being treated for a suspected CDI recurrence. Some subjects received up to 24 hours of standard CDI treatment prior to enrollment, which was subsequently determined to have an effect on baseline microbiota (see results). These subjects were studied separately and removed from the primary analysis.

### Stool DNA extraction

DNA was extracted using the QIAquick FAST DNA Stool kit (QIAGEN, Mississauga, Canada) with these modifications: ~250mg of frozen stool was suspended in 1.4mL of Inhibitex buffer (QIAGEN) supplemented with 50μg of lysozyme (Sigma) and 13.5U of lysostaphin (Sigma), then subjected to 5 minutes of bead-beating with 500mg of 0.1mm silica/zirconium beads (BioSpec Corp) on a Vortex Genie with a MoBio adapter. The supernatant was incubated at 70°C for 10 minutes with 100μg Proteinase K QIAGEN) and 400μg RNase A (Sigma), microfuged for 1 minute and transferred to a clean tube, after which the manufacturer’s protocol was resumed. DNA was eluted in 100μL of buffer. If the concentration as measured on a Nanodrop 2000 was <10μg/mL, the extraction was repeated. A 50μL portion of the extracted DNA was removed for qPCR analysis and the remainder was used for 16S rDNA amplicon generation.

### Quantitative PCR (qPCR) analysis

After ethanol precipitation of the extracted DNA to eliminate inhibitors, the reconstituted DNA was checked for purity and concentration using a Synergy H1 Hybrid Reader with Take3 Micro-Volume plates (BioTek, Winooski, VT) and then diluted with PCR-grade water to 5ng/μL. The DNA levels of bacterial groups were assessed using well-established PCR primers/conditions (S2 Table) [30–34]. Using the Mx3000P qPCR System (Agilent Technologies, Santa Clara, CA), qPCR was performed on each sample in triplicate in a final volume of 20μL containing 25ng DNA template, primers at 0.3μM, and SYBR Green 2x Master Mix (QIAGEN), with a FAM-tagged probe at 0.25μM for Eubacteria. Threshold cycle values were converted to copies per ng of DNA using a standard curve. Standards were prepared by performing PCR using the above group-specific primers on appropriate bacterial strains or DNA from normal stool. The products were cloned using Invitrogen Zero Blunt TOPO PCR Cloning Kit (ThermoFisher Scientific, Waltham, MA), and inserts were verified by sequencing at the Tufts University Core Facility. A Basic Local Alignment Search Tool (BLAST) search was performed to identify the closest matching database sequence (S3 Table). A range of 10-fold serially diluted plasmid standard DNA (5X10^8^ to 500 copies) was run on each qPCR plate in triplicate. Standard curve R^2^ values ranged from 0.990–0.999, with inter-assay variability and coefficient of variance below 6% and 0.07, respectively. Copies per gram of stool were then calculated, accounting for initial sample DNA concentrations and stool weights. The change in bacterial levels (Δlog_10_ copies/gram stool) from entry level to each available successive time-point was determined for each participant and median changes were then calculated.

### High-throughput sequencing

Amplicons of the V4 region of bacterial 16S rDNA were generated from extracted DNA using primers described by Caporaso [35]. Triplicate PCR amplifications on 50ng of DNA were performed using a 5’ HotStarTaq Master Mix kit (Thermo Fisher) with the following conditions: 94°C for 3 minutes followed by 35 cycles of 94°C for 45 seconds, 50°C for 60 seconds, and 72°C for 90 seconds, with a final extension of 72°C for 10 min. Amplicon DNA concentrations were determined by Quant-iT assay (Invitrogen, Carlsbad, CA), pooled in equimolar concentrations, purified (Qiaquick PCR Purification Kit, QIAGEN), and then eluted from Agencourt Ampure XP beads (Beckman-Coulter, Beverly, MA). Amplicon pools of ~100 samples each were sequenced on an Illumina MiSeq at the Tufts University Core Facility by a standard 250bp paired-end Illumina protocol. Initial data was processed using QIIME Version 1.8.0, an open- source software on the Galaxy website (http://huttenhower.sph.harvard.edu/galaxy/) [35, 36]. Operational taxonomic units (OTUs) were defined by 99% identity; taxonomic assignment was by closed reference with GreenGenes 13_8. To analyze differences between treatment groups and across timepoints, the OTU table was normalized to the lowest number of sequences per sample, then consolidated by summing to species level and to each successively higher taxonomic level. The alpha diversity parameters Chao and Shannon (based on number of OTUs) and Phylogentic Diversity (PD) (based on phylogenetic relationships), were generated in QIIME; alpha diversity is widely regarded as an indication of microbiota “health”. For beta diversity, which describes the relationships between samples, weighted Unifrac distance matrices were generated in QIIME.

### Statistical analyses

For both qPCR and high-throughput sequencing data, significance of differences between treatment groups at each time point was calculated using Mann-Whitney U test while within each treatment arm, significance of differences between time-points was assessed by the Wilcoxon Signed Rank test; both were performed with GraphPad Prism (GraphPad, San Diego, CA) on log-transformed data.

For high-throughput sequencing data, the LEfSe (Linear Effect Size) algorithm [37] was used to identify significant differences in microbiota composition between baseline and each study timepoint, followed by Wilcoxon Signed Rank tests on identified taxa. The Benjamini-Hochberg procedure was used to control the false discovery rate at 0.10 [38]. The MaAsLin algorithm on the Galaxy website was used to find associations of taxa with treatment. For beta diversity, principal co-ordinates analyses on weighted Unifrac distance matrices generated in QIIME were performed in the vegan package in the statistical program R. For qPCR data, to analyze whether trajectories across time differed between treatment groups, repeated measures models with time and group as categorical fixed factors [39] were used on log-transformed data, with diagnostics to assess the impact of potential influential points.

## Results

### Pre-enrollment standard CDI therapy resulted in different baseline microbiota

Twenty-two participants in the trial had received up to 24 hours of metronidazole and/or vancomycin immediately prior to randomization. The qPCR analysis of their baseline samples revealed that *C*. *coccoides* and *C*. *leptum* groups were 0.76 and 0.42 log_10_ copies/gram lower (p = 0.004 and 0.025, respectively) and Enterobacteriaceae was 1.28 log_10_ copies/gram higher (p = 0.018) than those who did not receive pre-enrollment treatment. Their baseline microbiota relative abundance profiles also differed dramatically (S1 Fig). Therefore, the pretreated participants were excluded from the primary qPCR and microbiota data analysis, leaving 22 participants in each treatment arm (Fig 1). There were no significant differences in age, sex, BMI, or use of PPIs, NSAIDS, opioids or probiotics between treatment groups.

### Baseline microbiota were not different between treatment groups

At study entry, there was no difference by qPCR in log_10_ copies/gram stool in any bacterial group between ridinilazole- and vancomycin-treated participants (Table 1; see S4 Table for supporting data). Study participants’ baseline samples had significantly lower copies/gram stool of all groups tested (p <0.5–<0.001) except Enterobacteriaceae, when compared to a population of 14 healthy volunteers as a benchmark.

**Table 1:** Comparison of bacterial group median quantities at each timepoint within treatment arms.

	Ridinilazole				Vancomycin				Controls
# of subjects→	n = 21				n = 18				n = 14
	**Baseline**	**Day 5**	**Δlog**_**10**_	**p-value**	**Baseline**	**Day 5**	**Δlog**_**10**_	**p-value**	
**Bacteroides**	6.15E+09	1.19E+10	0.28	**ns**	6.28E+09	1.02E+06	−3.47	<0.0001	1.07E+10
**Eubacteria**	5.48E+09	7.35E+09	0.08	**ns**	5.60E+09	1.59E+09	−0.43	0.016	1.77E+10
**Enterobacteriaceae**	6.98E+07	1.18E+08	0.02	**ns**	4.79E+07	3.61E+08	0.60	<0.001	5.95E+06
***C*. *coccoides***	9.14E+08	4.11E+08	−0.47	**ns**	1.40E+09	1.86E+06	−3.00	<0.0001	5.75E+09
***C*. *leptum***	5.83E+08	1.85E+08	−0.28	**ns**	2.67E+08	2.33E+05	−3.02	<0.0001	2.33E+09
***Prevotella***	2.95E+09	3.39E+09	0.06	**ns**	1.10E+09	5.52E+06	−2.30	<0.0001	1.02E+10
# of subjects→	n = 21				n = 19				
	**Baseline**	**Day 10**	**Δlog**_**10**_	**p-value**	**Baseline**	**Day 10**	**Δlog**_**10**_	**p-value**	
**Bacteroides**	6.15E+09	1.12E+10	0.09	**ns**	8.52E+09	1.14E+06	−3.42	<0.0001	
**Eubacteria**	5.48E+09	6.98E+10	0.01	**ns**	5.74E+09	1.81E+06	−0.53	0.006	
**Enterobacteriaceae**	6.98E+07	1.69E+08	−0.01	**ns**	3.38E+07	5.38E+08	0.51	<0.0001	
***C*. *coccoides***	9.14E+08	5.57E+08	−0.29	**ns**	1.48E+09	1.89E+06	−2.83	<0.0001	
***C*. *leptum***	5.83E+08	9.21E+07	−0.71	0.003	3.05E+08	2.41E+05	−2.94	<0.0001	
***Prevotella***	2.95E+09	5.01E+09	0.12	**ns**	1.16E+09	6.99E+06	−1.97	0.003	
# of subjects→	n = 18				n = 15				
	**Baseline**	**Day 25**	**Δlog**_**10**_	**p-value**	**Baseline**	**Day 25**	**Δlog**_**10**_	**p-value**	
**Bacteroides**	5.02E+09	5.82E+09	−0.01	**ns**	6.28E+09	9.98E+08	−0.05	**ns**	
**Eubacteria**	5.45E+09	7.18E+09	0.09	**ns**	5.63E+09	5.86E+09	0.05	ns	
**Enterobacteriaceae**	6.03E+07	5.18E+07	0.08	**ns**	1.15E+07	4.35E+08	1.03	0.017	
***C*. *coccoides***	9.04E+08	1.88E+09	0.16	**ns**	1.62E+09	1.07E+09	0.05	ns	
***C*. *leptum***	6.42E+08	5.61E+08	−0.04	**ns**	3.43E+09	3.80E+08	−0.36	ns	
***Prevotella***	1.31E+09	2.45E+09	0.10	**ns**	1.57E+09	5.72E+08	0.03	ns	
# of subjects→	n = 17				n = 10				
	**Baseline**	**EOS**	**Δlog**_**10**_	**p-value**	**Baseline**	**EOS**	**Δlog**_**10**_	**p-value**	
**Bacteroides**	6.15E+09	1.02E+10	0.15	**ns**	5.12E+09	9.77E+09	0.26	**ns**	
**Eubacteria**	5.48E+09	9.10E+09	0.01	**ns**	5.31E+09	6.70E+09	0.01	**ns**	
**Enterobacteriaceae**	5.08E+07	2.19E+07	0.26	**ns**	3.68E+07	1.31E+08	1.10	**ns**	
***C*. *coccoides***	7.28E+08	1.93E+09	0.36	**ns**	1.62E+09	2.72E+09	0.27	**ns**	
***C*. *leptum***	6.42E+08	5.22E+08	−0.11	**ns**	3.43E+08	1.77E+08	−0.78	**ns**	
***Prevotella***	1.63E+09	4.49E+09	0.14	**ns**	1.53E+09	3.45E+09	0.21	**ns**	

Medians calculated using subjects with samples at both timepoints, and for 14 controls.

Fold change expressed as log_10_ copies/gram.

Significance determined by Wilcoxon matched pairs signed rank test.

Controls = healthy volunteers as described in methods.

### Ridinilazole has minimal impact on microbiota group levels compared to vancomycin

Vancomycin-treated participants had a small but statistically significant loss in total bacterial biomass as measured by qPCR, with median decreases of 0.43 and 0.53 log_10_ copies/gram of stool for Eubacteria at D5 and D10, respectively (Table 1; see S4 Table for supporting data). This was not observed in ridinilazole-treated participants. Profound effects on *Bacteroides* group were seen in vancomycin-treated participants, with median decreases of > 3 log_10_ copies/gram stool at both D5 and D10 compared to baseline. Similar, statistically significant decreases were found in the *C*. *coccoides* and *C*. *leptum* groups and in *Prevotella* (3.00, 3.02, and 2.30 log_10_ copies/g at D5, and 2.83, 2.94, and 1.97 log_10_ copies/g at D10, respectively). In marked contrast, ridinilazole-treated participants had no significant differences in microbiota groups assessed at any time point, except for a modest decrease in *C*. *leptum* levels at D10 (−0.71 log_10_ copies/g; p = 0.05), which recovered completely by D25.

Conversely, vancomycin-treated participants had significant increases in Enterobacteriaceae which persisted beyond end of treatment, with a maximum increase of more than 1 log_10_ at D25 when compared to baseline. In ridinilazole-treated participants, no significant changes in Enterobacteriaceae were observed throughout the study.

**Fig 2:**
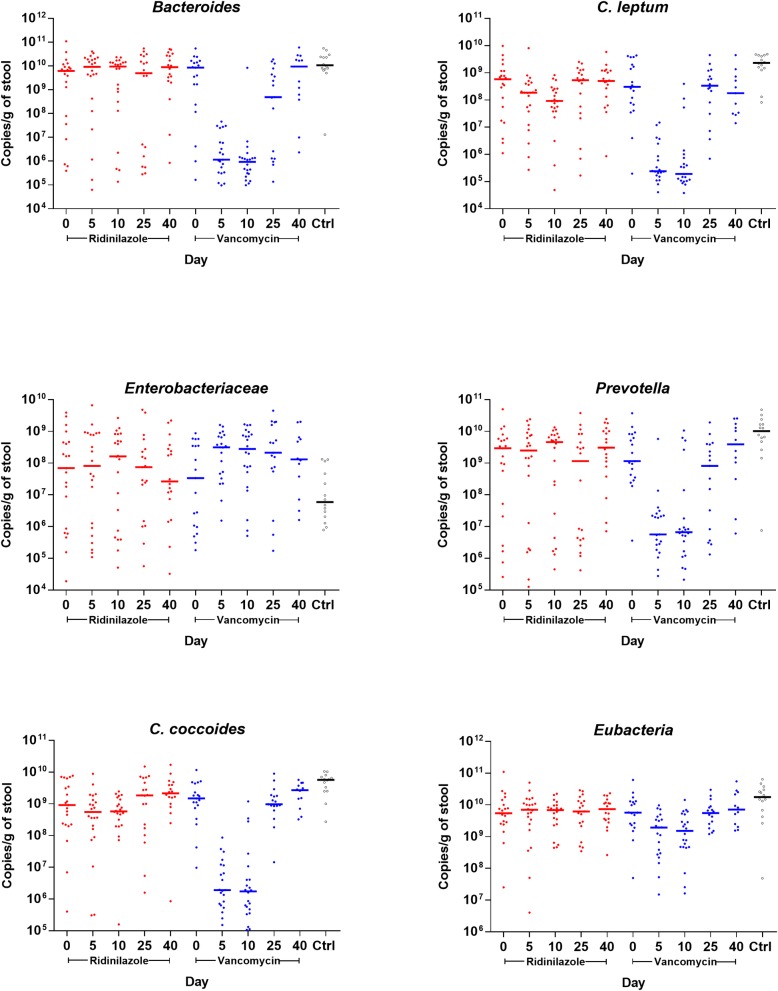
Microbiota levels belonging to different taxonomic groups measured by qPCR in samples from study participants and 14 healthy controls. Red circles represent participants treated with ridinilazole and blue circles represent participants treated with vancomycin. Bolded bars represent the median of all samples at that time point.

To test whether the trajectories across time for any microbiota group differed between treatments, p-values for the interaction of time and treatment group were calculated using repeated measures model on log-transformed data (Fig 2; see S4 Table for supporting data) from all participants at every time point. P-values were highly statistically significant for *Bacteroides*, *C*. *leptum*, Enterobacteriaceae, and *Prevotella* groups (all <0.0001), but not for the *C*. *coccoides* group (p = 0.06) or Eubacteria (p = 0.07). Exclusion of potential influential points did not change results.

### Ridinilazole has less impact on alpha and beta diversity than vancomycin

All high-throughput sequences generated in this study were submitted to the NCBI SRA site and accepted as study SRP110532. Taxonomy was assigned to a total of 10,757 OTUs. The OTU table was rarefied to 20,900 sequences per sample, resulting in a lower limit of detection (LLD) of a percent relative abundance of 0.00478%. Prior to analysis of differences in microbiota composition between samples, taxa which were present in <10% of samples were trimmed from the OTU table; the resulting table used in LEfSe analyses comprised 224 taxa levels.

As microbiota are disrupted at the time of CDI diagnosis, alpha diversity measurements are shown from healthy controls for comparison. As expected, baseline alpha diversity indices for study participants compared to healthy controls (Table 2) were significantly lower (p <0.0001) in all measured parameters. While both study antibiotics resulted in significant decreases in both OTU-based alpha diversity parameters by EOT, mean values were significantly lower following vancomycin than ridinilazole (range p <0.01 to p <0.0001). When the alpha diversity index phylogenetic diversity was considered, the difference between antibiotics was dramatic. Ridinilazole treatment resulted in no significant change in PD between baseline and EOT, whereas a major loss in diversity was observed with vancomycin (p >0.0001). Excluding those participants who experienced recurrence, effects on alpha diversity had resolved by day 25 following either treatment (data not shown).

**Table 2:** Comparison of alpha diversity measures between baseline and end-of-treatment (EOT) within each treatment arm (horizontal), and between treatment arms (vertical).

Alpha Diversity Measure	Baseline		EOT		
**Chao**	**Mean**	Stdev*	**Mean**	**Stdev**	p-value (Baseline:EOT)**
Control	1039	217.0			
Ridinilazole	485.6	188.6	387.7	108.7	<0.05
Vancomycin	490.8	164.3	295.5	99.0	<0.001
*p*-value (Rid:Vanco)***	NS		<0.0001		
**Shannon**	**Mean**	**Stdev**	**Mean**	**Stdev**	
Control	6.18	0.67			
Ridinilazole	4.43	1.40	3.98	1.08	<0.05
Vancomycin	3.96	1.14	3.11	0.92	<0.05
*p*-value (Rid:Vanco)	NS		<0.01		
**PD**	**Mean**	**Stdev**	**Mean**	**Stdev**	
Control	32.50	6.83			
Ridinilazole	14.26	4.87	12.09	3.58	NS
Vancomycin	14.66	3.79	7.70	2.15	<0.0001
*p*-value (Rid:Vanco)***	NS		<0.0001		

*Stdev = Standard deviation.

**Comparison of baseline to EOT for each drug treatment.

***Comparison of ridinilazole to vancomycin.

p-values (Rid:Vanco and Baseline:EOT) derived by Mann-Whitney test.

PD = Phylogenetic diversity.

**Fig 3:**
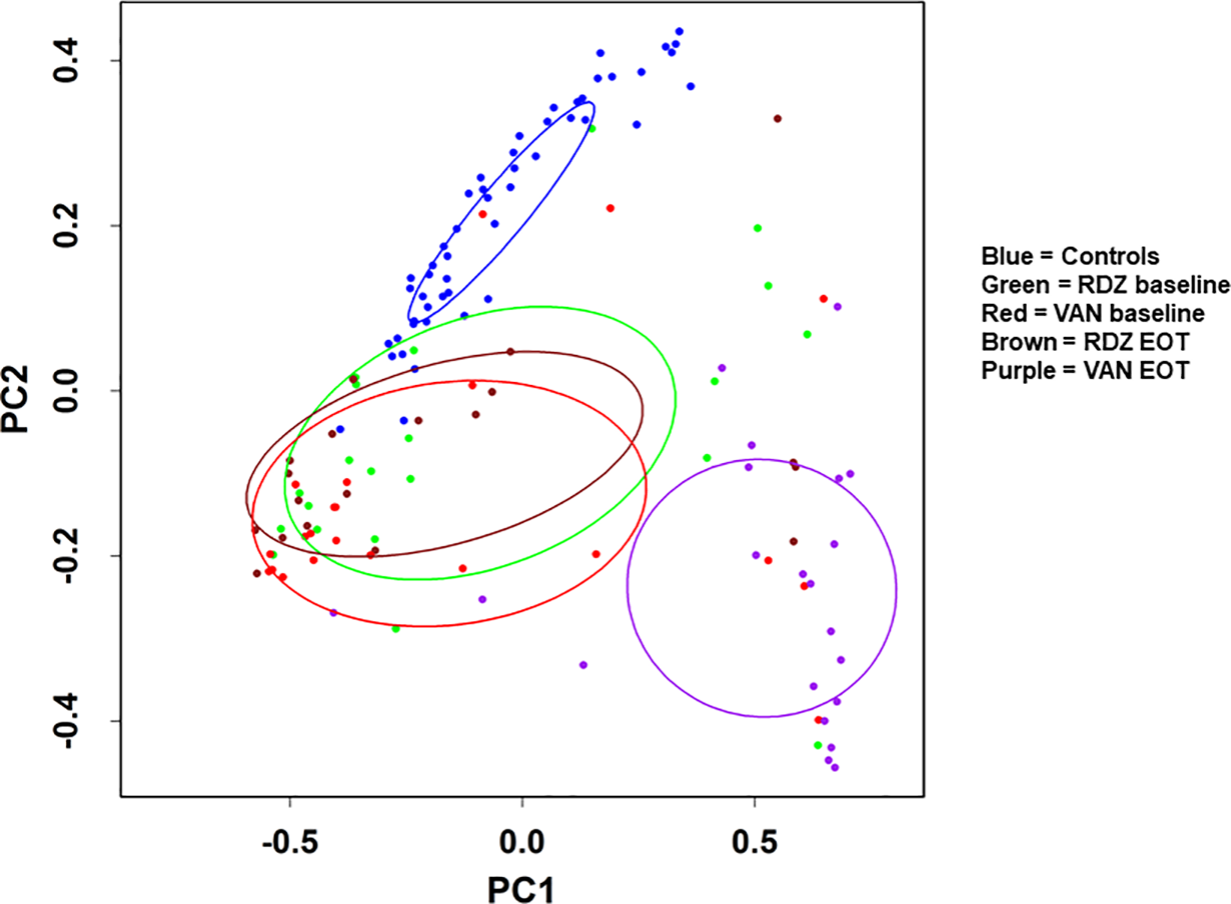
Beta diversity between participants receiving ridinilazole or vancomycin and normal controls. Principal co-ordinate analysis was performed between samples at baseline and EOT from participants receiving ridinilazole or vancomycin in the vegan package in R on weighted Unifrac distances generated in QIIME. For another comparator, healthy controls are also shown. Ellipses represent 95% confidence interval of each cluster. Abbreviations: RDZ, ridinilazole; VAN, vancomycin; PC1, first Principal co-ordinate; PC2, second principal co-ordinate.

Beta diversity analyses confirmed that study participants’ baseline microbiota were significantly different from controls, though not from each other (Fig 3). Following antibiotic treatment, the effect of ridinilazole on microbial community structure was minimal, while that of vancomycin was profound. The cluster of vancomycin-treated participants at EOT is significantly different from those at baseline, is further from controls, and demonstrates the deleterious impact on community structure by vancomycin; in contrast, the ridinilazole cluster was not significantly shifted during treatment.

### Taxonomic composition is modestly affected by ridinilazole compared to vancomycin

**Fig 4:**
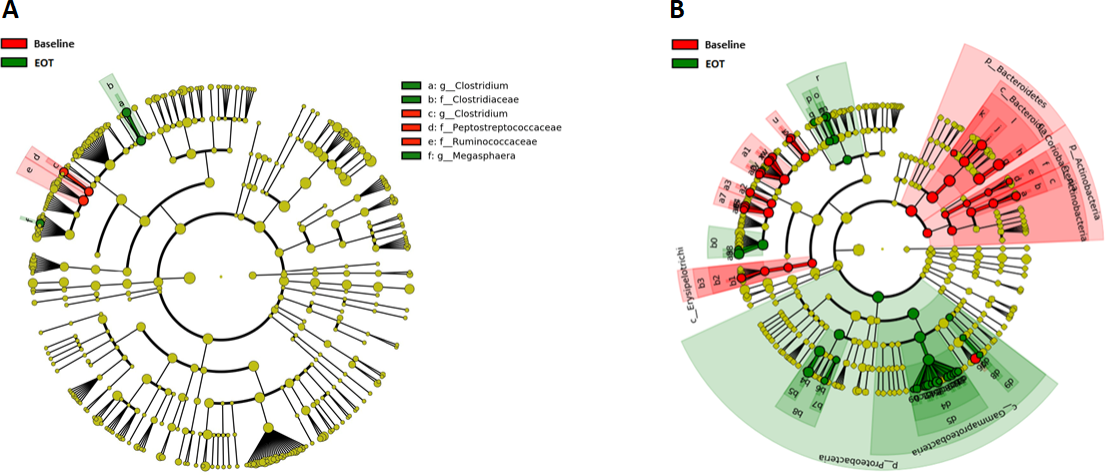
Effects of vancomycin and ridinilazole on relative abundance of taxa at baseline vs end-of-therapy. Cladograms were generated by LEfSe showing taxa with significantly higher relative abundance at baseline in red, and those with significantly higher relative abundance at end-of-therapy (EOT) in green. The phylogenetic tree is represented by concentric rings with phyla located at the innermost ring and subsequent taxonomic levels descend outwards to the species level. Panel A: Ridinilazole; Panel B: Vancomycin.

As in the qPCR analysis, the effect of study antibiotic on microbial composition was assessed by comparing samples at baseline to samples from the same participant at each available successive time point. Vancomycin had a wide-ranging effect (Fig 4), with 103 taxonomic designations identified by LEfSe as discriminating between baseline and day 10 at a linear discriminant analysis (LDA) value of 2. Although the change in abundance of the phylum Firmicutes was not significant, significant decreases were detected in four Firmicutes families by the Wilcoxon ranked sum test after controlling the false discovery rate using the Benjamini-Hochberg procedure. These families were: Peptostreptococcaceae (the family of which *C*. *difficile* is a member), Ruminococcaceae, Erysipelothrichaceae and Lachnospiraceae (Table 3). In the Lachnospiraceae and Ruminococcaceae families, abundances dropped 3 and 2 logs respectively, to below the lower limit of detection (LLD). Genera of interest within Lachnospiraceae that fell below the LLD were the butyrate-producing *Blautia*, *Coprococcus*, *Dorea*, *Roseburia*, and the species *Ruminococcus gnavus*, all associated with a “healthy” gut. In Ruminococcaceae, the genera *Oscillospira* and *Ruminococcus* also fell below the LLD. Concurrently, abundances of the genera *Veillonella* (family Veillonellaceae) and *Lactobacillus* (class Bacilli) increased by over a log.

**Table 3:** Taxa to the genus level with ≥2-fold change in relative abundance from baseline to end of treatment (EOT) with vancomycin or ridinilazole.

	Vancomycin				Ridinilazole			
Taxa	Baseline	EOT	Fold change	q value	Baseline	EOT	Fold change	q value
**Actinobacteria**	1.49E-04	9.57E-05	4.9	0.021				
c_Actinobacteria g_Bifidobacterium	2.14E-04	<LLD	4.5	0.029				
c_Coriobacteria f_Coriobacteriaceae	1.35E-04	<LLD	2.8	0.025				
**Bacteroidetes**	7.96E-01	4.49E-04	1773.5	0.006				
f_Bacteroidaceae g_Bacteroides	7.36E-01	1.17E-04	6274.8	0.008				
f_Porphyromonadaceae	5.42E-03	<LLD	113.5	0.034				
**Firmicutes**								
c_Bacilli f_Lactobacillaceae	4.79E-05	2.43E-03	50.8	0.019				
c_Clostridia f_Clostridiaceae g_Clostridium	2.39E-04	<LLD	5.0	0.027	9.57E-04	9.57E-05	10.0	0.002
c_Clostridia f_Lachnospiraceae	6.76E-02	<LLD	1415.8	0.003				
g_Blautia	7.59E-03	<LLD	158.9	0.004				
g_Coprococcus	6.55E-04	<LLD	13.7	0.007				
g_Dorea	4.98E-04	<LLD	10.4	0.016				
g_Roseburia	1.91E-04	<LLD	4.0	0.022				
g_Ruminococcus	3.23E-03	<LLD	67.6	0.014				
c_Clostridia f_Peptostreptococcaceae	8.55E-04	<LLD	17.9	0.005	1.53E-03	<LLD	35.8	<0.001
*Clostridium difficile*	1.91E-04	<LLD	4.0	0.026	7.18E-04	<LLD	15.0	0.001
c_Clostridia f_Ruminococcaceae	2.72E-02	<LLD	570.2	0.002	4.13E-02	2.82E-03	14.6	0.002
g_Faecalibacterium	1.46E-03	<LLD	30.5	0.013				
g_Oscillospira	2.14E-03	<LLD	44.8	0.015				
g_Ruminococcus	1.91E-04	<LLD	4.0	0.021				
c_Clostridia f_Veillonellaceae g_Veillonella	2.51E-03	3.05E-02	12.2	0.025				
c_Erysipelotrichi f_Erysipelotrichiaceae	2.22E-03	<LLD	46.6	0.005				
g_Eubacterium	8.13E-04	<LLD	17.0	0.022				
**Proteobacteria**	2.86E-02	6.55E-01	22.9	0.002				
f_Enterobacteriaceae	2.16E-03	4.73E-01	219.4	0.001				
g_Citrobacter	4.79E-05	1.23E-02	257.6	0.012				
g_Enterobacter	<LLD	7.60E-04	15.9	0.008				
g_Erwinia	<LLD	4.06E-04	8.5	0.010				
g_Escherichia	2.34E-04	1.30E-02	55.6	0.009				
g_Klebsiella	4.79E-05	0.05445	1137.6	0.010				
g_Salmonella	<LLD	3.58E-04	7.5	0.011				
g_Serratia	<LLD	5.74E-04	12.0	0.016				
g_Trabulsiella	<LLD	1.35E-04	2.8	0.027				
f_Pasteurellaceae g_Haemophilus	2.14E-04	<LLD	4.5	0.033				
	Decrease		Increase					
		<5 fold		<5 fold				
		>5 to <10 fold		>5 to <10 fold				
		>10 fold		>10 fold				
		>100 fold		>100 fold				
		>1000 fold		>1000 fold				

P-values were determined by the Wilcoxon rank sum test, following which q values were derived using the Benjamini-Hochberg procedure to hold the false discovery rate to 0.10

c = class, o = order, f = family, g = genus, LLD = lower limit of detection

In other phyla, the genus *Bifidobacterium* (phylum Actinobacteria) decreased to below the LLD, a reduction of over 4-fold. Mirroring our qPCR data, the most dramatic losses were in the Bacteroidetes, which was the most abundant phylum at baseline and dropped by over 3 logs, from 79% to 0.03% relative abundance. Simultaneously, a dramatic increase in Proteobacteria was observed, primarily in the family Enterobacteriaceae, where the relative abundance at EOT was 219 times higher than baseline. The median abundance of several taxa increased from undetectable to levels >10x LLD, including the pathogens *Citrobacter*, *Enterobacter*, *Serratia* and *Klebsiella*, with abundance of the latter expanding by over 3 logs. Univariate analysis in MaAsLin assessing associations of taxa with either treatment group at baseline and EOT was confirmatory of LEfSe results (S5 Table and S6 Table). The highest negative correlations with vancomycin were those of Bacteroides, Ruminococcaceae, and Lachnospiraceae and the highest positive correlation, that of Enterobacteriaceae.

Some differences persisted at D25, two weeks after end of treatment, with the Enterobacteriaceae *E*. *coli* and *Klebsiella* being 19- and 43-fold higher than baseline, although these differences were not significant when corrected for false discovery. These changes had resolved by EOS. The levels of *C*. *difficile* on days 25 and 40 were not significantly different from baseline.

In marked contrast, the antibacterial effect of ridinilazole was confined to the phylum Firmicutes (Fig 4, Table 3). By EOT, the median abundance of *C*. *difficile* was reduced to below the LLD and remained undetectable thereafter. Modest changes were detected in the Clostridiaceae and Ruminococcaceae families, with 10-and 14-fold decreases in relative abundance from baseline, respectively. By day 25, and at EOS the only changes from baseline were the sustained loss of detectable *C*. *difficile* and a 13-fold increase in the genus *Ruminococcus* in the family Ruminococcaceae (data not shown).

### Subjects with recurrence had both lower alpha diversity scores and taxonomic composition differences compared to those without recurrence

Due to the limited number (only 2) of ridinilazole-treated participants with recurrence in the primary analysis, analysis of differences between the treatment arms in the microbiota of participants with recurrence could not be performed. To explore factors associated with recurrence (or recurrence protection) we considered the status of the microbiota of the entire clinical study cohort (82 patients) at EOT. Alpha diversity of participants who had a subsequent recurrence was significantly lower at EOT in all three parameters measured than that of those who did not experience recurrence (Table 4). Lachnospiraceae and Ruminococcaceae were higher in those who did not experience recurrence, while several genera of Enterobacteriaceae were higher in those who did (S2 Fig).

**Table 4:** Differences at end of treatment in alpha diversity and median relative abundance of taxa between subjects who had or did not have a confirmed recurrence of *C*. *difficile* during the study.

**Alpha diversity**		Recurrence	No recurrence	P-value
	n	11	69	
**Chao**	Mean	280	365	<0.05
	Stdev*	81.6	133	
**Shannon**	Mean	2.89	3.67	<0.05
	Stdev	0.89	1.11	
**PD**	Mean	7.33	10.56	<0.01
	Stdev	1.04	3.94	
**Median Relative Abundance**		Recurrence	No recurrence	*P*-value
Taxa higher in subjects without recurrence				
Firmicutes		3.18E-02	1.11E-01	<0.05
o_Clostridiales		1.57E-02	8.85E-02	<0.05
f_Lachnospiraceae		<LLD	2.68E-03	<0.05
g_Blautia		<LLD	1.91E-04	<0.05
f_Ruminococcaceae		<LLD	6.70E-04	<0.05
Taxa higher in subjects with recurrence				
Proteobacteria				
c_Gammaproteobacteria		5.70E-01	1.60E-01	<0.05
f_Enterobacteriaceae		5.70E-01	1.05E-01	<0.05
g_Erwinia		5.74E-04	9.57E-05	<0.05
g_Klebsiella		7.57E-02	5.74E-03	<0.05
*Klebsiella oxytoca*		3.11E-03	9.57E-05	<0.05
g_Salmonella		5.26E-04	<LLD	<0.01
*Salmonella enterica*		5.26E-04	<LLD	<0.01

*Stdev = standard deviation.

PD = phylogenetic diversity.

c = class, o = order, f = family, g = genus, s = species.

## Discussion

Our observations regarding vancomycin’s effects on the intestinal microbiota are in accord with those of previous studies [11, 40]. We found that vancomycin treatment resulted in a biomass decrease of *Bacteroides* and *Prevotella* groups, and that of both Firmicutes subgroups we examined, as well as a concomitant increase in the biomass of the Enterobacteriaceae group. High-throughput sequencing showed numerous changes following vancomycin, including decreased alpha diversity parameters, significant changes in beta diversity and decreased relative abundance of *Bifidobacterium*, two Bacteroidetes families and four Firmicutes families, as well as increases in Enterobacteriaceae. These alterations in microbial community structure have been associated with susceptibility to CDI (16–20). Utilizing fluorescent in situ hybridization/flow cytometry, Tannock et al observed a similar decrease in commensal clostridial species following vancomycin treatment, with concomitant outgrowth of Enterobacteriaceae [11]. Louie et al conducted a qPCR-based study of the effects of fidaxomicin and vancomycin treatment of CDI on quantities of major microbiota [31]. Interestingly, they found persistent *Bacteroides* decreases several weeks after completion of vancomycin treatment compared to fidaxomicin-treated samples. Although our qPCR analysis did not detect a delayed recovery, our high-throughput sequencing was suggestive of this, also revealing the significant persistence of Enterobacteriaceae.

This novel finding of this study is the limited disruption of intestinal microbiota following ridinilazole treatment, both in terms of biomass (how many are there) and composition (which taxonomic groups are represented). Both are likely important in preventing *C*. *difficile* colonization, disease, and recurrence, by preserving sufficient density of the correct type(s) of species to create an environment not conducive to *C*. *difficile* expansion. Loss of alpha diversity was significantly lower with ridinilazole than with vancomycin, indicating a smaller impact on microbiota health and preservation of colonization resistance. This was confirmed by the absence of an expansion of the phylum Proteobacteria. The only microbiota biomass loss was with the *C*. *leptum* group at D10, with this loss being modest compared to that seen with vancomycin. While high-throughput sequencing revealed significant decreases in some *Clostridiales* taxa other than *C*. *difficile*, this was not unexpected, as ridinilazole shows some effect on total colony counts of Clostridia species [41]. The changes in Clostridiaceae and Ruminococcaceae were lower than those resulting from vancomycin and resolved with cessation of antibiotic. The Lachnospiraceae, known to enhance colonic epithelial cell health and immune function, in part by generating the short-chain fatty acid butyrate, were unaffected by ridinilazole. In contrast to findings in vancomycin-treated participants, the median relative abundance of *C*. *difficile* in ridinilazole-treated participants fell below LLD by D5, remaining there until EOS.

In sum, ridinilazole is a promising, effective CDI antimicrobial with minimal effects on bystander indigenous colonic flora. Its microbiota-preserving narrow-spectrum activity is likely responsible for the observed decreased recurrence rate compared to vancomycin treatment.

## Supporting information

S1 Fig Effect of treatment of CDI in 24-hour period prior to participant study enrollment. Cladogram generated by LefSe showing taxa in baseline samples with a significantly higher percent relative abundance when comparing participants who did (Yes = green) or did not (No = red) receive anti-CDI treatment within 24 hours of study enrollment. The phylogenetic tree is represented by concentric rings, with phyla at the innermost ring and lower taxonomic levels in the rings tiered successively outwards.
(TIF)

S2 Fig Comparison of taxa at end-of-therapy from participants who did and did not experience recurrence. Cladogram generated by LefSe showing taxa at end-of-therapy samples with a significantly higher percent relative abundance when comparing participants who did (green) or who did not (red) recur. The phylogenetic tree is represented by concentric rings, with phyla at the innermost ring and lower taxonomic levels in the rings tiered successively outwards.
(TIF)

S1 Table Institutional Review Boards (IRBs) by study center. (DOCX)

S2 Table DNA primers used for qPCR to quantify changes in major components of microbiota during and after treatment of *Clostridium difficile* infection. (DOCX)

S3 Table Sequences of plasmid standards for qPCR. (DOCX)

S4 Table qPCR results for 6 bacterial groups for all available timepoints for each subject. The mean and median values are calculated only for those subjects who did not receive anti-CDI antibiotic prior to enrollment.
(PDF)

S5 Table Taxa significantly and negatively associated with vancomycin treatment at EOT. (DOCX)

S6 Table Taxa significantly and positively associated with vancomycin treatment at EOT. (DOCX)
