# Supplementary figures and images for: Enhanced preservation of the human intestinal microbiota by ridinilazole, a novel Clostridium difficile-targeting antibacterial, compared to vancomycin
Source: PLoS One. 2018 Aug 2;13(8):e0199810. doi: 10.1371/journal.pone.0199810 (PMC6071993; doi:10.1371/journal.pone.0199810)

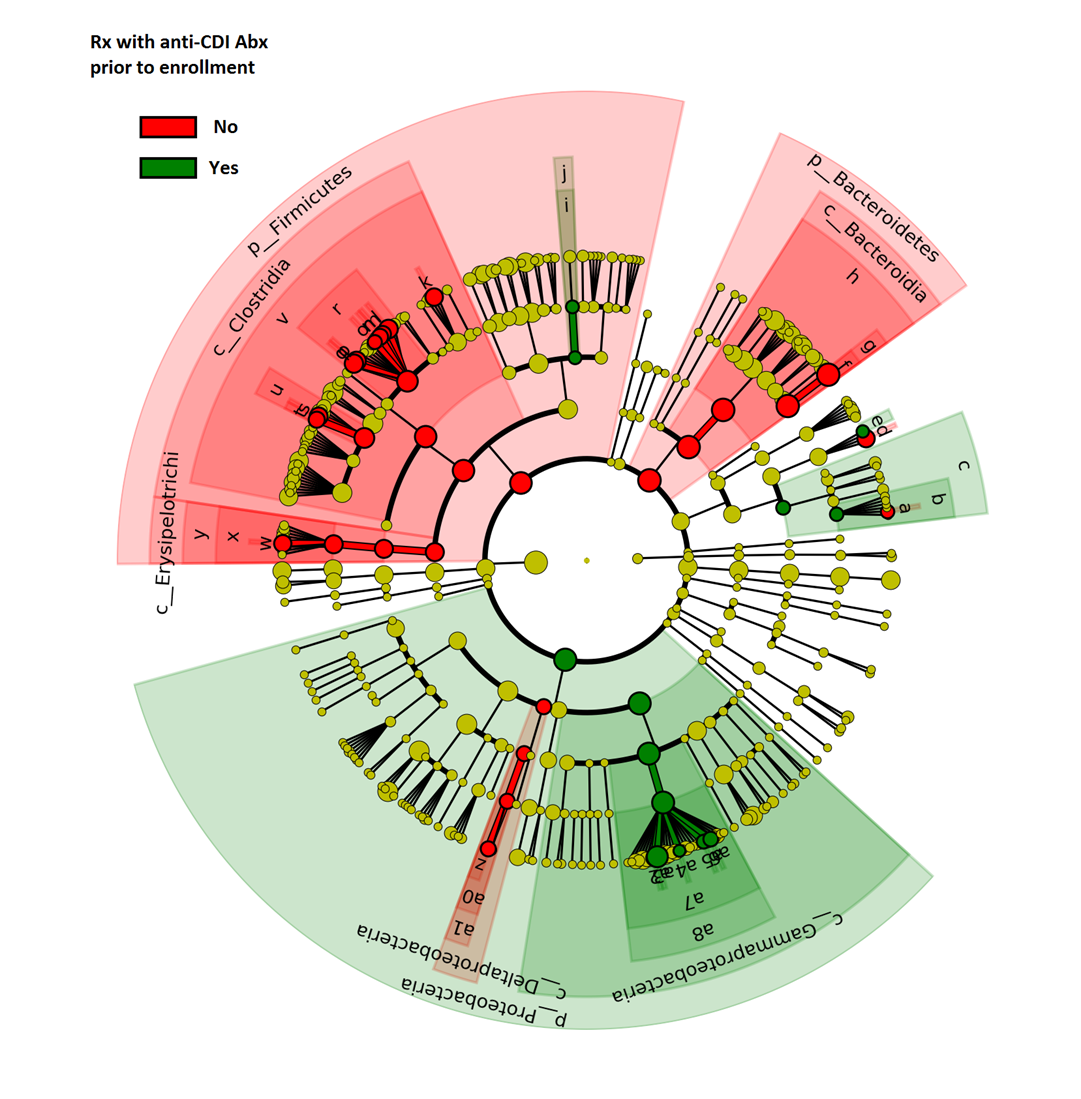

Supplement: S1 Fig — Cladogram generated by LefSe showing taxa in baseline samples with a significantly higher percent relative abundance when comparing participants who did (Yes = green) or did not (No = red) receive anti-CDI treatment within 24 hours of study enrollment. The phylogenetic tree is represented by concentric rings, with phyla at the innermost ring and lower taxonomic levels in the rings tiered successively outwards. (TIF) [file pone.0199810.s001.tif]

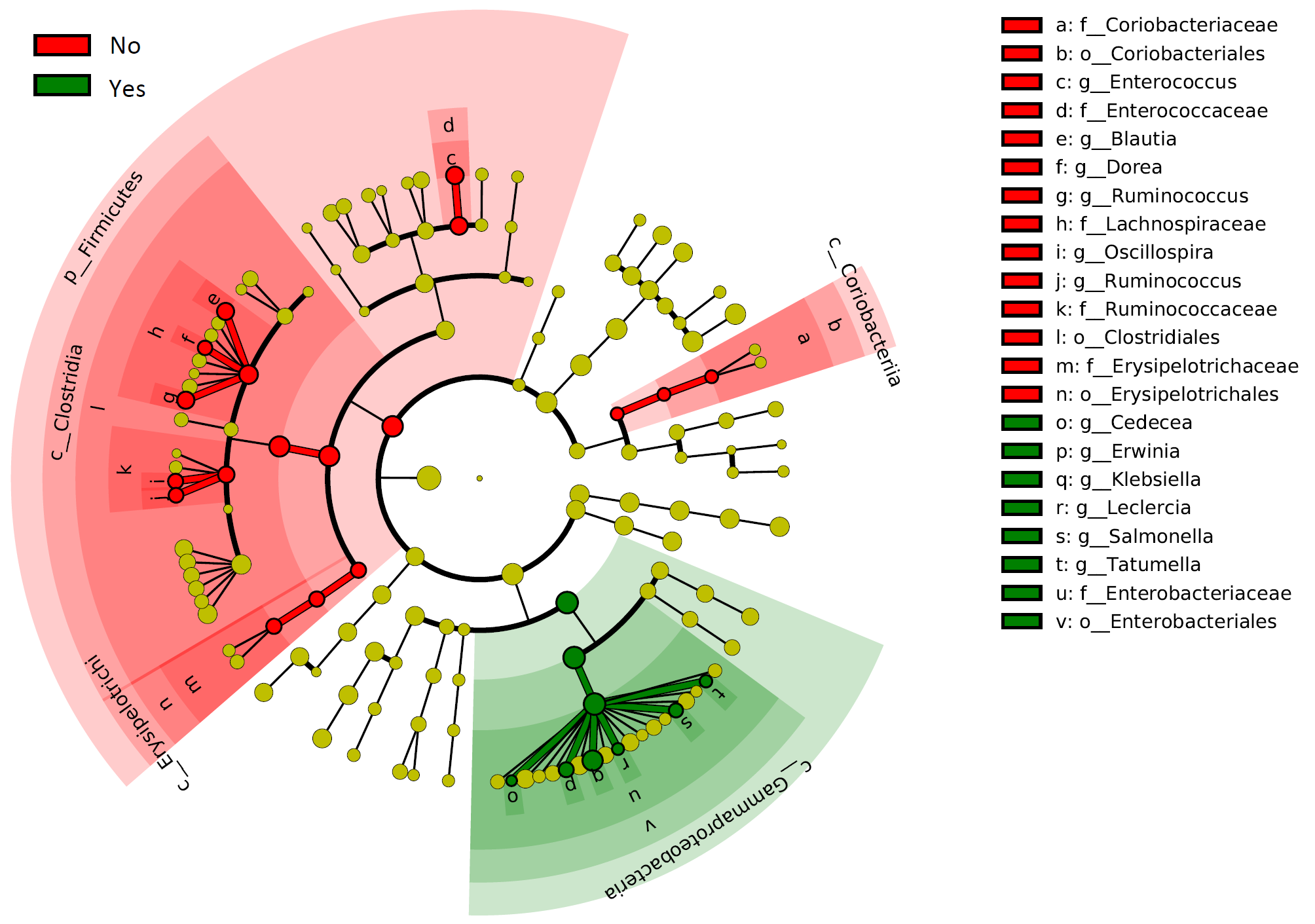

Supplement: S2 Fig — Cladogram generated by LefSe showing taxa at end-of-therapy samples with a significantly higher percent relative abundance when comparing participants who did (green) or who did not (red) recur. The phylogenetic tree is represented by concentric rings, with phyla at the innermost ring and lower taxonomic levels in the rings tiered successively outwards. (TIF) [file pone.0199810.s002.tif]
