# Supplementary material for: Enhanced preservation of the human intestinal microbiota by ridinilazole, a novel Clostridium difficile-targeting antibacterial, compared to vancomycin
Source: PLoS One. 2018 Aug 2;13(8):e0199810. doi: 10.1371/journal.pone.0199810 (PMC6071993; doi:10.1371/journal.pone.0199810)
Supplement: S1 Table — (DOCX) [file pone.0199810.s003.docx]

**S1 Table**

| Site# | PI Last name | IRB/EC |
| --- | --- | --- |
| 002 | Pullman | Copernicus Group IRB |
| 003 | Bhan | Henry Ford Health Systems Institutional Review Board |
| 004 | Weiss | Hopital Maisonneuve-Rosemont/Centre affilie a l'Universite de Montreal |
| 006 | Campbell | CHESAPEAKE IRB |
| 007 | Baird | Western Institutional Review Board |
| 009 | Lucasti | Copernicus Group IRB |
| 010 | Lee | Hamilton Integrated Research Ethics Board (HIREB) |
| 011 | Tan | Summa Health System |
| 012 | Golan | Western Institutional Review Board |
| 014 | Mullane | AURA IRB |
| 016 | Chopra | Wayne State University IRB |
| 017 | Talan | Olive View UCLA Education & Research Institute |
| 018 | Schrock | North Memorial IRB |
| 019 | Nathan | Copernicus Group IRB |
| 021 | Philips | North Mississippi Health Services IRB |
| 022 | Gordon | VA Long Beach Healthcare System |
| 024 | Oughton | Bureau D'Ethique de la Recherche |
| 026 | Riska | BRANY IRB |
| 027 | Young | University of Minnesota IRB |
| 029 | Maliakkal | Western Institutional Review Board |
| 030 | Yacyshyn | Copernicus Group IRB |
| 031 | Kamepalli | St Rita's Health Partners |
| 032 | Siegel | Copernicus Group IRB |
| 033 | Deck | Memorial Health Services IRB |
| 035 | Saberhagen | Copernicus Group IRB |
| 036 | Zenilman | Johns Hopkins Medicine IRB |
| 037 | Steinberg | Copernicus Group IRB |
| 038 | Pesant | Centre de sante et de services sociaux de Saint-Jerome |
| 039 | Khanna | Copernicus Group IRB |
| 041 | Minang | Essentia Health IRB |
| 042 | Tamang | Copernicus Group IRB |
| 044 | Welton | Copernicus Group IRB |
| 045 | Wang | Western Institutional Review Board |
