# Supplementary material for: Enhanced preservation of the human intestinal microbiota by ridinilazole, a novel Clostridium difficile-targeting antibacterial, compared to vancomycin
Source: PLoS One. 2018 Aug 2;13(8):e0199810. doi: 10.1371/journal.pone.0199810 (PMC6071993; doi:10.1371/journal.pone.0199810)
Supplement: S2 Table — (DOCX) [file pone.0199810.s004.docx]

**S2 Table**

| **Group** | **Primer name** | **Sequence** | **Reference** |
| --- | --- | --- | --- |
| *Bacteroides* | Bac303F  Bac708R | GAA GGT CCC CCA CAT TG  CAA TCG GAG TTC TTC GTG | Bernhard et al, 2000 (30) |
| *Clostridium coccoides* | Erec482R  Eub338F | GCT TCT TAG TCA RGT ACC G  ACT CCT ACG GGA GGC AGC | Louie et al, 2012 (31) |
| *Clostridium leptum* | Sg-clept-F  Sg-clept-R | GCA CAA GCA GTG GAG T  CTT CCT CCG TTT GTC AA | Matsuki et al, 2002 (32) |
| Enterobacteriaceae | Eco1457F  Eco1652 | CAT TGA CGT TAC CCG CAG AAG AAG C  CTC TAC GAG ACT CAA GCT TGC | Bartosch et al, 2004 (33) |
| Eubacteria | 8F  515R  338P | AGT TTG ATC CTG GCT CAG  GWA TTA CCG CGG CKG CTG  FAM GCT GCC TCC CGT AGG AGT BHQ1 | Jiang et al, 2009 (34) |
| *Prevotella* | CFB286F  CFB719R | GTA GGG GTT CTG AGA GGA  AGC TGC CTT CGC AAT CGG | Louie et al, 2012 (31) |

FAM=6-carboxyfluorescein; BHQ1=Black Hole Quencher 1
