# Supplementary material for: Enhanced preservation of the human intestinal microbiota by ridinilazole, a novel Clostridium difficile-targeting antibacterial, compared to vancomycin
Source: PLoS One. 2018 Aug 2;13(8):e0199810. doi: 10.1371/journal.pone.0199810 (PMC6071993; doi:10.1371/journal.pone.0199810)
Supplement: S3 Table — (DOCX) [file pone.0199810.s005.docx]

**S3 Table**

| **Primer Set** | **Referenced Organism** | **Length** | **Match** | **Sequence** |
| --- | --- | --- | --- | --- |
| **Bacteroides** | Bacteroides sp. HGA0257 16S ribosomal RNA gene, partial sequence | 419 | 99% | **GAAGGTCCCCCACATTG**GAACTGAGACACGGTCCAAACTCCTACGGGAGGCAGCAGTGAGGAATATTGGTCAATGGGCGAGAGCCTGAACCAGCCAAGTAGCGTGAAGGATGACTGCCCTATGGGTTGTAAACTTCTTTTATAAAGGAATAAAGTCGGGTATGGATACCCGTTTGCATGTACTTTATGGATAAGGATCGGCTAACTCCGTGCCAGCAGCCGCGGTAATACGGAGGATCCGAGCGTTATCCGGATTTATTGGGTTTAAAGGGAGCGTAGATGGATGTTTAAGTCAGTTGTGAAAGTTTGCGACTCAACCGTAAAATTGCAGTTGATACTGGATATCTTGAGTGCAGTTGAGGCAGGCGGAATTCGTGGTGTAGCGGTGAAATGCTTAGATAT**CACGAAGAACTCCGATTG** |
| ***Clostridium coccoides*** | *Blautia coccoides* strain DSM 29138 16S ribosomal RNA gene, partial sequence | 139 | 95% | **ACTCCTACGGGAGGCAGC**AGTGGGGAATATTGCACAATGGGGGAAACCCTGATGCAGCAACGCCGCGTGAGTGAAGAAGTATTTCGGTATGTAAAGCTCTATCAGCAGGAAAGAAAATGA**CGGTACCTGACTAAGAAGC** |
| ***Clostridium leptum*** | *Clostridium leptum* 16S rRNA gene, strain DSM 753T *Clostridium leptum* ATCC 29065 (taxid:428125) | 241 | 90% | **GCACAAGCAGTGGAGTAT**GTGGTTTAATTCGAAGCAACGCGAAGAACCTTACCAGGTCTTGACATCGAGTGACGAACATAGAGATATGTTCTTCCTTCGGGACACGAAGACAGGTGGTGCATGGTTGTCGTCAGCTCGTGTCGTGAGATGTTGGGTTAAGTCCCGCAACGAGCGCAACCCTTATCATTAGTTGCTACGCAAGAGCACTCTAATGAGACTGCCG**TTGACAAAACGGAGGAAG** |
| **Entero-bacteriaceae** | *Escherichia coli* JJ1887, complete genome | 190 | 100% | **CATTGACGTTACCCGCAGAAGAAGC**ACCGGCTAACTCCGTGCCAGCAGCCGCGGTAATACGGAGGGTGCAAGCGTTAATCGGAATTACTGGGCGTAAAGCGCACGCAGGCGGTTTGTTAAGTCAGATGTGAAATCCCCGGGCTCAACCTGGGAACTGCATCTGATACTG**GCAAGCTTGAGTCTCGTAGAG** |
| **Eubacteria** | *Escherichia coli str*. K-12 substr. MG1655 strain K-12 16S ribosomal RNA, complete sequence (EUBACTERIA) | 517 | 100% | **AGTTTGATCATGGCTCAG**ATTGAACGCTGGCGGCAGGCCTAACACATGCAAGTCGAACGGTAACAGGAAGAAGCTTGCTTCTTTGCTGACGAGTGGCGGACGGGTGAGTAATGTCTGGGAAACTGCCTGATGGAGGGGGATAACTACTGGAAACGGTAGCTAATACCGCATAACGTCGCAAGACCAAAGAGGGGGACCTTCGGGCCTCTTGCCATCGGATGTGCCCAGATGGGATTAGCTAGTAGGTGGGGTAACGGCTCACCTAGGCGACGATCCCTAGCTGGTCTGAGAGGATGACCAGCCACACTGGAACTGAGACACGGTCCAGACTCCTACGGGAGGCAGCAGTGGGGAATATTGCACAATGGGCGCAAGCCTGATGCAGCCATGCCGCGTGTATGAAGAAGGCCTTCGGGTTGTAAAGTACTTTCAGCGGGGAGGAAGGGAGTAAAGTTAATACCTTTGCTCATTGACGTTACCCGCAGAAGAAGCACCGGCTAACTCCGTGC**CAGCAGCCGCGGTAATAC** |
| **Prevotella** | Prevotella denticola gene for 16S ribosomal RNA, partial sequence, strain: JCM 8528 | 446 | 86% | **GTAGGGGTTCTGAGAGGA**AGGTCCCCCACATTGGAACTGAGACACGGTCCAAACTCCTACGGGAGGCAGCAGTGAGGAATATTGGTCAATGGGCGAGAGCCTGAACCAGCCAAGTAGCGTGAAGGATGACTGCCCTATGGGTTGTAAACTTCTTTTATAAAGGAATAAAGTCGGGTATGGATACCCGTTTGCATGTACTTTATGAATAAGGATCGGCTAACTCCGTGCCAGCAGCCGCGGTAATACGGAGGATCCGAGCGTTATCCGGATTTATTGGGTTTAAAGGGAGCGTAGATGGATGTTTAAGTCAGTTGTGAAAGTTTGCGGCTCAACCGTAAAATTGCAGTTGATACTGGATATCTTGAGTGCAGTTGAGGCAGGCGGAATTCGTGGTGTAGCGGTGAAATGCTTAGATATCACGAAGAACT**CCGATTGCGAAGGCAGCT** |
