# Supplementary material for: Enhanced preservation of the human intestinal microbiota by ridinilazole, a novel Clostridium difficile-targeting antibacterial, compared to vancomycin
Source: PLoS One. 2018 Aug 2;13(8):e0199810. doi: 10.1371/journal.pone.0199810 (PMC6071993; doi:10.1371/journal.pone.0199810)
Supplement: S4 Table — The mean and median values are calculated only for those subjects who did not receive anti-CDI antibiotic prior to enrollment. (PDF) [file pone.0199810.s006.pdf]

Bacteroides

CDI Rx 24 hrs prior to enrollment	Pretreatment Abx	Study drug	Subject ID#	Baseline (day 1)	Day 5	End of treatment (day 10)	Day 25	End of study (day 40)	Sample taken at time of suspected recurrence (REC)	Recurrence confirmed (yes/no)
Y	Vancomycin (V)	Ridinilazole (R)	002-013	1.04E+11	2.33E+10	4.67E+10	3.94E+10	2.30E+10		
Y	Metrinidazole (M)	R	007-002	1.51E+05	8.49E+03	1.53E+04	1.07E+05	1.35E+05		
Y	V+M	R	009-002	3.11E+09	2.19E+06	1.66E+09	1.04E+07	2.62E+07		
Y	M	R	017-003	1.72E+10	1.13E+10	2.06E+10		1.46E+10		
Y	M	R	022-001		1.63E+10	2.41E+10	9.27E+09	1.01E+09		
Y	V+M	R	031-002	1.47E+10		6.29E+10	2.86E+06		1.96E+07	Yes
Y	M	R	033-008	9.35E+09	5.09E+06	2.33E+06	2.74E+05	9.74E+09	3.12E+05	No
Y	M	R	033-011	3.82E+06	7.02E+06	2.40E+10	3.97E+10	8.23E+09		
Y	V+M	R	038-001		3.20E+05	3.50E+09	2.28E+10	2.55E+10		
Y	V	R	038-004	1.28E+06	3.35E+05	1.85E+10	4.75E+10			
Y	V	R	042-003		6.79E+09	5.99E+09	1.99E+10	2.75E+09		
Y	M	R	045-003	5.55E+05	2.40E+06	2.60E+06	1.15E+06	6.52E+05		
N		R	002-007	1.29E+09	1.65E+05	1.10E+09	3.89E+06	1.28E+07		
N		R	002-009	6.15E+09	4.27E+08	3.15E+08	3.92E+09	7.68E+09		
N		R	002-012	1.82E+10	4.06E+10	2.26E+10				
N		R	009-005	7.46E+05	6.23E+04	1.38E+05	5.88E+05	2.72E+10		
N		R	009-007	8.44E+06	1.88E+10	7.76E+09	3.14E+10	2.36E+09		
N		R	019-001	3.61E+07	1.27E+08	1.31E+08	1.60E+06	8.53E+05		
N		R	019-003	2.15E+09	1.19E+10	1.37E+10	6.66E+09	3.66E+09	9.74E+09	No
N		R	019-006	3.88E+09	4.37E+09	1.12E+10	1.24E+10	2.63E+10		
N		R	019-007	3.83E+10	1.97E+10	1.76E+10	4.10E+10	3.31E+10		
N		R	019-009	6.87E+09	1.48E+10	1.69E+10	2.56E+10	3.75E+10		
N		R	021-001	7.81E+09	4.38E+09	1.45E+10	5.08E+06	1.09E+10	4.11E+09	
N		R	029-002	1.28E+10	6.37E+09	1.64E+09	1.39E+10	2.02E+09	1.24E+10	no
N		R	030-005	9.17E+09	2.29E+10	1.20E+10	3.82E+09	3.99E+08		
N		R	031-001	8.83E+09	1.34E+10	1.15E+10				
N		R	032-003	4.61E+09	1.78E+10	2.26E+06				yes
N		R	033-009	7.83E+07	3.44E+10	1.40E+10	5.49E+10	5.09E+10		
N		R	035-001	7.14E+09	2.64E+10	1.42E+10	2.97E+10	4.82E+10		
N		R	036-001	3.98E+05	2.16E+07	4.18E+05	2.84E+05	1.70E+10		
N		R	037-004	1.17E+10	2.22E+10	7.63E+09	1.53E+10	1.02E+10		
N		R	037-008	6.21E+05	1.17E+06	4.73E+05	5.56E+05			
N		R	037-014		4.68E+09	8.23E+08	3.11E+05	3.36E+09		
N		R	037-015	1.08E+11	3.78E+09	2.33E+10	4.97E+09	4.54E+09		
			**Median**	**6.15E+09**	**9.12E+09**	**9.47E+09**	**4.97E+09**	**8.92E+09**	**4.11E+09**	
			**Mean**	**1.17E+10**	**1.21E+10**	**8.68E+09**	**1.28E+10**	**1.59E+10**		
			**# of Samples**	**21**	**22**	**22**	**19**	**18**	**5**	

CDI Rx 24 hrs prior to enrollment	Pretreatment Abx	Study drug	Sample ID#	Baseline (day 1)	Day 5	End of treatment (day 10)	Day 25	End of study (day 40)	Sample taken at time of suspected recurrence	Recurrence confirmed (yes/no)
Y	M	Vancomycin (V)	002-014	2.08E+06	1.73E+06	1.38E+05				
Y	V	V	007-001	1.60E+10	1.41E+07	6.96E+04	9.67E+06	7.93E+09		
Y	M	V	009-010	6.42E+09	5.30E+07	7.38E+06	1.35E+06	1.67E+09		
Y	M	V	019-004	1.24E+10	6.98E+06	4.19E+07	7.67E+05	4.74E+05		
Y	M	V	029-001	8.94E+07	4.77E+04	3.45E+04	1.06E+06	2.64E+04		yes
Y	M	V	029-004	3.49E+09	4.55E+05	1.54E+05	2.14E+09	5.43E+09		
Y	M	V	033-007	3.46E+08	8.67E+09	4.08E+09	2.41E+09			
Y	V+M	V	033-010	6.70E+09	3.89E+05	2.27E+06	4.28E+05	1.26E+06	3.44E+06	yes
Y	M	V	035-003	4.27E+09	3.94E+07	3.77E+07	5.35E+06	8.69E+08	2.55E+10	no
Y	V+M	V	045-001	1.08E+09	1.89E+05	1.13E+05		8.86E+09		
N		V	002-004	2.53E+10	1.07E+06	4.22E+05	1.26E+06		6.06E+05	yes
N		V	002-011	2.37E+08	3.37E+05	1.46E+05	4.65E+08	1.22E+09		
N		V	002-015	8.90E+09	2.30E+06	7.23E+05			1.36E+06	Yes
N		V	002-016	4.20E+06	6.21E+06	1.48E+06				yes
N		V	009-001	1.64E+05	3.33E+05	2.94E+05	1.36E+05	1.00E+07		
N		V	009-003	1.17E+08	3.04E+07	1.18E+06	4.38E+09	2.23E+09	1.21E+10	no
N		V	009-004	4.03E+09	1.21E+05	1.16E+05	3.59E+09			
N		V	009-006	8.52E+09	3.07E+05	9.74E+04	2.62E+06	2.34E+06		
N		V	017-002	1.71E+09	5.91E+06	1.32E+06	7.93E+09	2.82E+10		
N		V	019-005	1.42E+10	7.64E+05	1.25E+06	1.27E+10	1.81E+10		
N		V	019-008	1.64E+10	3.27E+06	6.72E+06	1.53E+09	1.73E+10		
N		V	019-010		4.60E+07	4.50E+05	2.37E+09	1.66E+10		
N		V	019-013	2.40E+10	5.52E+05	8.38E+09	1.31E+06	5.97E+10		
N		V	019-014	1.69E+09	1.14E+05	3.13E+05	1.64E+08	3.83E+08		
N		V	021-003	1.33E+10		3.99E+06	1.89E+10		2.70E+10	Yes
N		V	030-004	5.48E+10	2.50E+07	2.20E+06		2.69E+10		
N		V	035-002	1.05E+10	9.77E+05	1.14E+06			1.23E+06	Yes
N		V	037-006		1.94E+06	4.74E+05	7.07E+05			yes
N		V	037-010	9.86E+05	9.68E+04	1.56E+05	9.52E+05			no
N		V	037-016		1.15E+06	1.38E+06	4.90E+08	6.28E+08	2.27E+08	no
N		V	042-001	3.57E+09	2.79E+07	1.35E+06				no
N		V	042-002	1.65E+10	3.05E+07	2.25E+05	1.54E+10			
			**Median**	**8.52E+09**	**1.15E+06**	**9.32E+05**	**4.90E+08**	**9.43E+09**	**1.14E+08**	
			**Mean**	**1.07E+10**	**8.82E+06**	**3.82E+08**	**3.99E+09**	**1.43E+10**	**6.55E+09**	
			**# of Samples**	**19**	**21**	**22**	**17**	**12**	**6**	

Eubacteria

Study drug	Subject ID#	Baseline (day 1)	Day 5	End of treatment (day 10)	Day 25	End of study (day 40)	Sample taken at time of suspected recurrence (REC)
Ridinilazole (R)	002-013	7.84E+10	1.25E+10	3.07E+10	3.59E+10	2.75E+10	
R	007-002	7.10E+08	7.80E+04	5.90E+05	2.83E+09	6.35E+08	
R	009-002	8.05E+09	6.26E+08	5.46E+10	2.32E+09	1.22E+10	
R	017-003	2.27E+10	1.04E+10	1.45E+10		1.38E+10	
R	022-001		1.32E+10	1.58E+10	3.33E+09	5.24E+08	
R	031-002	1.26E+10		3.74E+10	6.58E+07		5.69E+09
R	033-008	5.45E+09	1.38E+09	9.67E+06			
R	033-011	2.93E+07	2.02E+07	1.36E+10	3.09E+10	8.17E+08	
R	038-001		2.46E+08	9.24E+09	1.90E+10	1.86E+10	
R	038-004	1.21E+07	5.01E+07	2.25E+07	3.52E+08		
R	042-003		6.35E+09	4.93E+09			
R	045-003	6.88E+08	3.16E+09	1.26E+10	1.66E+10	7.74E+09	
R	002-007	1.42E+09	5.05E+07	2.24E+10	4.91E+08	5.60E+09	
R	002-009	7.83E+09	4.45E+08	4.45E+08	5.12E+09	1.10E+10	
R	002-012	2.74E+10	5.02E+10	1.25E+10			
R	009-005	7.54E+09	3.59E+08	3.52E+09	9.36E+09	1.13E+10	
R	009-007	6.25E+08	1.65E+10	6.03E+09	1.14E+10	3.32E+09	
R	019-001	6.68E+09	9.80E+09	6.79E+09	4.52E+08	1.56E+09	
R	019-003	2.41E+09	1.02E+10	1.45E+10	6.21E+09	1.99E+09	1.85E+10
R	019-006	4.92E+09	3.44E+09	8.32E+09	8.14E+09	1.48E+10	
R	019-007	2.31E+10	7.35E+09	7.60E+09	2.16E+10	2.01E+10	
R	019-009	1.01E+10	5.73E+09	5.47E+09	1.33E+10	1.95E+10	
R	021-001	7.56E+09	4.25E+09	9.59E+09	3.47E+08	9.10E+09	4.76E+09
R	029-002	1.77E+10	1.02E+10	2.46E+09	2.89E+10	2.81E+09	1.06E+10
R	030-005	5.41E+09	1.09E+10	7.87E+09	2.14E+09	2.66E+08	
R	031-001	1.43E+10	2.85E+10	2.05E+10			
R	032-003	2.56E+09	1.11E+10	4.67E+08			

R	033-009	2.52E+07	1.57E+10	7.75E+09	2.71E+10	1.18E+10	
R	035-001	2.87E+09	6.75E+09	6.98E+09	1.80E+10	2.33E+10	
R	036-001	5.48E+09	4.06E+06	5.52E+08	2.97E+09	1.10E+10	
R	037-004	4.53E+09	5.42E+09	2.28E+09	5.52E+09	3.65E+09	
R	037-008	3.08E+09	1.50E+10	3.85E+09	1.33E+10		
R	037-014		1.62E+09	6.44E+08	6.61E+08	5.36E+09	
R	037-015	1.10E+11	2.51E+09	9.88E+09	2.71E+09	3.65E+09	
	**Median**	**5.48E+09**	**7.05E+09**	**6.88E+09**	**6.21E+09**	**7.35E+09**	**8.15E+09**
	**Mean**	**1.27E+10**	**9.83E+09**	**7.29E+09**	**9.36E+09**	**8.89E+09**	**1.13E+10**
	**# of Samples**	**21**	**22**	**22**	**19**	**18**	**3**
V	002-014	1.37E+09	2.37E+09	6.92E+08			
V	007-001	1.50E+10	5.27E+08	2.67E+08	3.20E+09	6.21E+09	
V	009-010	1.96E+10	2.00E+09	1.82E+09	3.24E+09	4.07E+09	
V	019-004	6.86E+09	1.32E+09				
V	029-001	6.22E+07	7.12E+06	2.12E+06	5.61E+08	1.30E+07	
V	029-004	3.34E+09	7.63E+08	2.22E+09	1.08E+10	2.22E+10	
V	033-007	1.59E+08	3.78E+09	1.57E+09	1.09E+09		
V	033-010	2.30E+09	2.16E+08	3.61E+08			5.59E+09
V	035-003	8.05E+09	6.07E+08	1.56E+09			2.90E+10
V	045-001	1.26E+09	1.55E+07	2.63E+07		8.58E+09	
V	002-004	1.72E+10	5.27E+07	7.06E+07	1.21E+09		6.43E+08
V	002-011	2.69E+09	1.12E+09	7.91E+08	4.27E+09	1.55E+09	
V	002-015	1.22E+10	1.47E+08	4.68E+08			4.32E+09
V	002-016	2.59E+09	7.25E+09	5.06E+09			
V	009-001	4.82E+09	2.18E+09	2.11E+09	3.00E+09	5.16E+09	
V	009-003	7.71E+08	4.27E+09	1.81E+09	4.89E+09	1.63E+09	6.61E+09
V	009-004	1.50E+09	2.75E+08	4.69E+08	5.55E+09		
V	009-006	5.80E+09	1.51E+07	1.63E+07	2.53E+09	2.49E+09	
V	017-002	2.30E+09	1.93E+09	6.00E+08	6.61E+09	2.54E+10	
V	019-005	1.08E+10	3.00E+08	2.56E+07	6.17E+09	8.24E+09	
V	019-008	9.88E+09	6.75E+08	7.76E+08	8.24E+09	9.34E+09	
V	019-010		3.40E+09	4.46E+08	1.52E+09	2.16E+10	

V	019-013	2.44E+10	1.25E+09	6.18E+09	1.52E+10	5.54E+10	
V	019-014	5.00E+07	2.19E+08	2.87E+09	1.34E+09	2.03E+09	
V	021-003	1.30E+10		1.44E+10	1.93E+10		2.52E+10
V	030-004	6.11E+10	3.44E+09	2.92E+09		2.85E+10	
V	035-002	5.74E+09	1.95E+09	1.22E+09			6.95E+09
V	037-006		4.98E+09	6.92E+09	1.83E+09		
V	037-010	1.31E+10	7.79E+09	6.36E+09	9.08E+09		
V	037-016		3.87E+08	4.77E+08	6.99E+09	5.94E+09	3.08E+10
V	042-001	1.91E+09	3.14E+09	5.31E+09			
V	042-002	5.45E+09	9.70E+09	2.44E+09	2.99E+10		
	**Median**	**5.74E+09**	**1.93E+09**	**1.51E+09**	**5.55E+09**	**7.09E+09**	**6.78E+09**
	**Mean**	**1.03E+10**	**2.59E+09**	**2.80E+09**	**7.51E+09**	**1.39E+10**	**1.24E+10**
	**# of Samples**	**19**	**21**	**22**	**17**	**12**	**6**

Enterobacteriaceae

Study drug	Subject ID#	Baseline (day 1)	Day 5	End of treatment (day 10)	Day 25	End of study (day 40)	Sample taken at time of suspected recurrence (REC)
Ridinilazole (R)	002-013	2.56E+09	1.52E+09	1.91E+08	1.40E+10	6.00E+06	
R	007-002	7.14E+08	8.49E+03	1.18E+04	7.33E+05	1.26E+07	
R	009-002	1.04E+09	6.28E+05	9.10E+08	8.71E+07	1.95E+09	
R	017-003	9.91E+07	1.91E+06	1.32E+07		5.34E+05	
R	022-001		3.78E+08	1.13E+07	6.63E+07	3.33E+07	
R	031-002	5.39E+05		1.16E+06	2.74E+04		1.27E+06
R	033-008	5.82E+07	1.54E+09	6.93E+06			1.83E+06
R	033-011	1.20E+09	9.18E+08	1.17E+09	1.60E+08	6.51E+08	
R	038-001		2.11E+05	1.44E+06	2.15E+06	9.56E+07	
R	038-004	9.68E+05	2.10E+08	1.35E+08	2.83E+06		
R	042-003		2.12E+08	1.81E+06			
R	045-003	4.49E+09	9.55E+08	7.75E+08	1.86E+08	9.31E+07	
R	002-007	4.49E+08	1.48E+05	4.99E+08	2.90E+07	8.08E+08	
R	002-009	1.81E+07	1.79E+07	3.85E+05	2.91E+05	2.31E+05	
R	002-012	3.92E+09	8.71E+08	8.17E+08			
R	009-005	2.96E+09	7.72E+08	1.26E+09	3.90E+09	1.88E+09	
R	009-007	1.90E+04	1.09E+05	5.09E+04	5.63E+04	3.27E+04	
R	019-001	1.91E+08	1.65E+09	4.81E+08	5.64E+08	2.21E+09	
R	019-003	1.65E+08	9.08E+08	1.59E+08	4.83E+09	1.42E+06	2.15E+07
R	019-006	6.30E+05	1.18E+08	1.33E+08	1.49E+06	1.63E+07	
R	019-007	6.00E+05	3.05E+05	1.77E+05	1.05E+06	1.61E+06	
R	019-009	9.94E+08	8.96E+08	4.28E+08	1.58E+08	2.19E+07	
R	021-001	4.56E+05	1.85E+05	7.45E+05	2.28E+08	1.74E+08	1.18E+07
R	029-002	8.89E+06	2.32E+08	3.34E+06	8.07E+07	3.74E+08	2.67E+08
R	030-005	1.55E+05	5.11E+05	5.18E+07	2.76E+07	1.20E+07	
R	031-001	8.65E+05	9.03E+05	3.93E+05			
R	032-003	4.63E+08	9.38E+08	4.01E+08			

R	033-009	1.59E+09	6.64E+09	1.06E+09	7.69E+08	1.89E+08	
R	035-001	6.98E+07	2.93E+07	1.13E+07	1.00E+06	7.38E+06	
R	036-001	1.69E+08	3.86E+07	8.27E+08	7.46E+07	1.28E+07	
R	037-004	2.43E+07	3.21E+08	1.69E+08	2.09E+07	2.81E+08	
R	037-008	4.06E+08	7.87E+08	2.65E+09	2.45E+07		
R	037-014		1.27E+06	4.56E+05	2.76E+08	3.12E+07	
R	037-015	5.08E+07	4.61E+07	8.59E+08	1.42E+08	2.38E+08	
	**Median**	**6.98E+07**	**8.19E+07**	**1.64E+08**	**7.46E+07**	**2.65E+07**	**1.18E+07**
	**Mean**	**5.47E+08**	**6.49E+08**	**4.46E+08**	**5.86E+08**	**3.47E+08**	
	**# of Samples**	**21**	**22**	**22**	**19**	**18**	

V	002-014	3.52E+06	6.33E+07	7.83E+05			
V	007-001	8.26E+08	8.39E+08	4.15E+08	1.65E+09	4.40E+08	
V	009-010	2.62E+09	7.11E+08	8.87E+08	3.62E+08	3.87E+07	
V	019-004	1.01E+09	2.63E+09	3.56E+09			
V	029-001	6.68E+08	4.49E+08	2.67E+08	1.03E+09		
V	029-004	3.62E+07	8.30E+05	2.59E+08	3.52E+07	7.52E+07	
V	033-007	3.20E+08	3.02E+08	1.67E+08	6.84E+08		
V	033-010	7.38E+09	1.84E+08	1.03E+08			1.39E+09
V	035-003	6.32E+08	4.12E+08	5.46E+08			6.70E+08
V	045-001	1.32E+05	9.20E+03	2.67E+04		6.89E+07	
V	002-004	1.08E+06	2.28E+07	7.55E+07	2.12E+08		5.20E+08
V	002-011	5.80E+08	1.42E+09	6.89E+08	1.59E+09	1.93E+09	
V	002-015	1.26E+08	3.13E+08	1.60E+09			1.48E+09
V	002-016	2.63E+06	1.15E+08	5.21E+07			
V	009-001	6.20E+07	2.13E+08	1.99E+08	7.50E+07	7.16E+06	
V	009-003	2.79E+06	9.94E+08	5.38E+08	6.90E+07	1.39E+08	7.81E+07
V	009-004	3.53E+08	3.41E+08	7.47E+08	2.00E+09		
V	009-006	1.81E+05	6.55E+06	1.74E+07	4.42E+08	2.01E+09	
V	017-002	8.72E+08	6.86E+08	9.55E+08	5.21E+08	3.16E+06	
V	019-005	3.48E+08	8.93E+08	1.12E+09	2.07E+09	6.53E+08	
V	019-008	5.93E+05	2.22E+07	5.10E+05	4.28E+08	9.53E+07	
V	019-010		4.63E+07	7.98E+07	1.73E+05	4.75E+08	

V	019-013	3.11E+05	4.03E+08	2.17E+08	1.56E+08	1.22E+08	
V	019-014	1.15E+07	3.80E+08	8.01E+08	1.73E+08	5.66E+08	
V	021-003	9.74E+05		1.60E+06	1.93E+09		1.44E+09
V	030-004	3.46E+08	1.60E+09	1.55E+09		3.78E+07	
V	035-002	3.38E+07	5.27E+07	1.35E+07			4.71E+08
V	037-006		1.54E+06	7.46E+05	5.54E+05		
V	037-010	5.95E+08	7.74E+08	1.67E+09	4.47E+09		
V	037-016		3.30E+07	8.15E+07	5.45E+07	1.61E+06	9.79E+06
V	042-001	4.95E+05	3.11E+08	3.42E+08			
V	042-002	5.70E+08	6.50E+08	6.99E+08	1.52E+06		
	**Median**	**3.38E+07**	**3.13E+08**	**2.79E+08**	**2.12E+08**	**1.30E+08**	**4.95E+08**
	**Mean**	**2.06E+08**	**4.42E+08**	**5.21E+08**	**8.36E+08**	**5.03E+08**	**6.67E+08**
	**# of Samples**	**19**	**21**	**22**	**17**	**12**	**6**

C. coccoides

Study drug	Subject ID#	Baseline (day 1)	Day 5	End of treatment (day 10)	Day 25	End of study (day 40)	Sample taken at time of suspected recurrence (REC)
Ridinilazole (R)	002-013	3.08E+09	1.22E+08	6.67E+08	2.98E+08	2.15E+09	
R	007-002	3.29E+06	1.39E+04	1.80E+04	5.16E+05	6.64E+08	
R	009-002	6.38E+08	1.90E+06	4.40E+08	4.90E+08	3.74E+09	
R	017-003	3.21E+09	1.70E+09	2.47E+09		3.10E+09	
R	022-001		9.78E+08	1.66E+09	1.11E+09	2.46E+08	
R	031-002	2.78E+09		4.93E+09	1.55E+05		2.66E+07
R	033-008	4.44E+08	2.36E+08	4.75E+06			7.53E+05
R	033-011	7.16E+05	3.38E+05	5.87E+07	3.05E+09	2.32E+09	
R	038-001		9.48E+05	4.67E+09	8.34E+09	3.35E+09	
R	038-004	5.23E+06	2.32E+07	8.23E+05	1.56E+08		
R	042-003		2.77E+08	2.44E+08			
R	045-003	5.55E+05	2.75E+05	6.14E+09	9.23E+09	3.48E+09	
R	002-007	2.21E+08	3.20E+05	1.94E+08	1.70E+08	1.58E+09	
R	002-009	4.55E+08	1.06E+07	9.39E+07	9.80E+08	1.93E+09	
R	002-012	3.10E+09	4.08E+09	2.47E+09			
R	009-005	3.48E+09	2.06E+08	1.79E+09	7.51E+09	9.00E+09	
R	009-007	6.78E+07	1.82E+09	1.59E+09	1.89E+09	2.94E+09	
R	019-001	2.41E+08	3.63E+08	9.71E+08	1.60E+06	8.53E+05	
R	019-003	1.98E+08	7.32E+07	5.57E+08	1.03E+08	5.09E+08	1.87E+09
R	019-006	1.08E+09	3.70E+08	5.99E+08	2.75E+09	2.46E+09	
R	019-007	6.36E+09	1.12E+09	8.60E+08	6.82E+09	4.88E+09	
R	019-009	6.79E+09	2.02E+09	2.07E+09	7.23E+09	3.91E+09	
R	021-001	1.97E+09	4.11E+08	1.37E+09	5.38E+06	7.96E+08	1.83E+09
R	029-002	7.68E+09	1.93E+09	5.00E+08	1.51E+10	1.65E+09	4.04E+09
R	030-005	2.25E+08	9.16E+08	4.66E+08	2.26E+08	2.47E+08	
R	031-001	9.14E+08	6.93E+08	1.97E+08			
R	032-003	4.06E+05	3.06E+05	1.60E+05			

R	033-009	6.97E+06	7.50E+08	7.67E+08	4.64E+09	5.35E+09	
R	035-001	5.82E+08	1.62E+08	2.13E+08	1.86E+09	1.70E+10	
R	036-001	7.08E+09	8.96E+07	5.15E+08	1.80E+09	1.01E+09	
R	037-004	7.28E+08	3.63E+08	7.20E+07	2.81E+09	3.94E+09	
R	037-008	1.19E+09	8.90E+09	1.91E+09	6.58E+09		
R	037-014		1.32E+09	6.62E+08	6.07E+07	2.37E+09	
R	037-015	7.07E+09	9.59E+08	3.17E+08	1.01E+09	1.66E+09	
	**Median**	**9.14E+08**	**5.52E+08**	**5.78E+08**	**1.86E+09**	**2.15E+09**	**1.83E+09**
	**Mean**	**2.35E+09**	**1.21E+09**	**8.27E+08**	**3.24E+09**	**3.40E+09**	**2.58E+09**
	**# of samples**	**21**	**22**	**22**	**19**	**18**	**3**
V	002-014	6.09E+06	1.55E+07	2.47E+08			
V	007-001	9.17E+08	1.06E+06	2.23E+05	4.98E+08	2.06E+09	
V	009-010	4.13E+06	9.30E+05	1.37E+06	1.90E+09	8.50E+08	
V	019-004	1.62E+09	2.41E+06	9.32E+06			
V	029-001	2.68E+07	3.30E+06	7.75E+04	2.49E+08	5.70E+05	
V	029-004	2.05E+08	9.56E+05	3.78E+07	4.55E+09	6.05E+09	
V	033-007	1.96E+07	1.73E+08	4.63E+07	1.52E+08		
V	033-010	3.14E+08	1.15E+06	2.95E+05	1.86E+05	7.13E+05	1.66E+09
V	035-003	1.06E+09	4.21E+06	1.24E+07	7.64E+05	1.69E+08	5.12E+09
V	045-001	3.70E+08	1.69E+05	2.51E+05		3.09E+09	
V	002-004	4.38E+09	5.35E+05	5.75E+05	9.06E+08		8.05E+08
V	002-011	1.82E+09	6.32E+05	3.32E+05	5.46E+09	4.59E+09	
V	002-015	5.18E+09	4.02E+06	1.89E+06			5.65E+08
V	002-016	2.27E+08	8.70E+07	2.68E+06			
V	009-001	1.75E+09	1.36E+06	1.13E+05	1.83E+08	7.02E+08	
V	009-003	9.68E+06	1.23E+07	1.94E+06	8.89E+08	3.25E+08	7.94E+08
V	009-004	3.35E+08	2.46E+05	1.07E+05	1.18E+09		
V	009-006	1.48E+09	1.53E+05	1.33E+05	1.89E+09	3.11E+09	
V	017-002	1.13E+09	1.91E+06	8.69E+05	2.90E+09	3.73E+09	
V	019-005	1.89E+09	8.33E+05	4.75E+05	9.67E+08	4.63E+09	
V	019-008	1.31E+09	1.57E+06	4.11E+06	1.29E+09	3.92E+08	
V	019-010		6.47E+06	3.57E+05	3.36E+08	5.73E+09	

V	019-013	2.31E+09	1.97E+06	3.48E+08	9.00E+09	2.84E+09	
V	019-014	9.12E+08	3.84E+05	4.06E+06	8.64E+08	1.52E+09	
V	021-003	4.76E+09		1.08E+07	8.53E+08		1.33E+10
V	030-004	1.17E+10	1.72E+07	1.20E+09		2.60E+09	
V	035-002	4.26E+07	1.16E+07	2.72E+07			1.82E+05
V	037-006		3.10E+07	2.28E+06	6.22E+08		
V	037-010	4.81E+09	5.83E+06	2.80E+08	1.46E+07		
V	037-016		7.12E+05	7.31E+05	1.71E+09	1.46E+09	2.78E+09
V	042-001	1.11E+09	3.79E+07	1.62E+06			
V	**Median**	**1.62E+09**	**1.94E+06**	**1.89E+06**	**9.36E+08**	**2.72E+09**	**7.99E+08**
	**Mean**	**2.51E+09**	**1.12E+07**	**8.98E+07**	**1.82E+09**	**2.64E+09**	**3.05E+09**
	**# of samples**	**18**	**20**	**21**	**16**	**12**	**6**

C. leptum

Study drug	Subject ID#	Baseline (day 1)	Day 5	End of treatment (day 10)	Day 25	End of study (day 40)	Sample taken at time of suspected recurrence (REC)
Ridinilazole (R)	002-013	8.71E+08	9.15E+07	2.21E+08	6.21E+07	1.51E+08	
R	007-002	3.03E+05	1.72E+04	1.37E+04	1.07E+05	1.35E+05	
R	009-002	1.51E+09	6.89E+05	6.64E+07	3.77E+08	1.86E+09	
R	017-003	8.42E+08	5.94E+07	1.38E+08		1.47E+09	
R	022-001		7.90E+08	9.92E+08	6.43E+08	2.30E+08	
R	031-002	6.57E+07		2.47E+08	2.74E+04		9.85E+05
R	033-008	1.43E+08	1.04E+07	3.56E+06			5.05E+05
R	033-011	3.32E+05	3.38E+05	1.31E+08	6.72E+08	3.87E+09	
R	038-001		1.28E+05	1.34E+08	4.27E+08	8.95E+07	
R	038-004	7.21E+05	1.45E+07	3.67E+05	9.47E+06		
R	042-003		7.90E+07	2.51E+07			
R	045-003	5.55E+05	1.70E+05	1.18E+09	1.70E+08	7.22E+07	
R	002-007	6.89E+08	2.70E+05	5.51E+07	1.74E+08	1.02E+09	
R	002-009	1.16E+09	1.21E+07	5.06E+07	1.24E+09	1.22E+09	
R	002-012	3.04E+09	8.01E+09	6.14E+08			
R	009-005	3.48E+08	7.96E+05	2.63E+08	1.30E+09	1.33E+08	
R	009-007	1.75E+07	3.99E+08	5.40E+08	4.08E+08	1.31E+09	
R	019-001	1.11E+06	2.51E+06	3.10E+06	1.60E+06	8.53E+05	
R	019-003	5.61E+07	5.48E+07	1.84E+08	2.14E+07	3.58E+07	3.36E+08
R	019-006	3.90E+08	3.63E+08	7.64E+07	4.99E+08	3.69E+08	
R	019-007	7.81E+08	1.85E+08	5.64E+07	8.49E+08	5.22E+08	
R	019-009	4.47E+09	1.42E+08	2.25E+08	2.49E+09	1.96E+09	
R	021-001	7.68E+08	2.30E+08	8.26E+08	6.91E+05	4.76E+08	7.27E+08
R	029-002	2.07E+09	4.97E+08	9.21E+07	2.20E+09	6.05E+08	1.02E+09
R	030-005	1.47E+07	3.79E+07	2.30E+07	3.28E+07	5.23E+07	
R	031-001	1.21E+08	6.37E+07	3.93E+05			
R	032-003	2.67E+06	1.91E+08	4.93E+04			

R	033-009	3.74E+06	2.02E+08	1.04E+08	6.40E+08	2.71E+08	
R	035-001	5.83E+08	4.38E+08	2.58E+08	1.51E+09	5.80E+09	
R	036-001	6.42E+08	7.77E+06	3.86E+07	5.73E+07	6.35E+07	
R	037-004	1.16E+09	1.84E+08	3.81E+07	5.86E+08	1.18E+09	
R	037-008	2.88E+08	8.01E+08	1.42E+08	5.35E+08		
R	037-014		1.82E+08	9.12E+07	1.67E+05	4.57E+08	
R	037-015	9.75E+09	6.55E+08	2.92E+08	9.91E+08	1.20E+09	
	**Median**	**5.83E+08**	**1.85E+08**	**9.16E+07**	**5.35E+08**	**4.99E+08**	**3.36E+08**
	**Mean**	**1.25E+09**	**5.76E+08**	**1.81E+08**	**7.12E+08**	**9.27E+08**	**6.96E+08**
	**# of samples**	**21**	**22**	**22**	**19**	**18**	**3**

V	002-014	1.79E+05	3.55E+05	3.82E+06			
V	007-001	1.46E+09	3.08E+05	6.96E+04	1.16E+07	1.71E+09	
V	009-010	2.10E+08	1.92E+07	1.03E+07	7.01E+08	8.11E+08	
V	019-004	1.53E+09	1.58E+06	1.58E+06	1.66E+07	2.03E+08	
V	029-001	3.70E+06	2.06E+06	3.45E+04	1.51E+07	2.64E+04	
V	029-004	3.29E+08	5.25E+04	1.54E+05	6.32E+08	7.36E+08	
V	033-007	3.63E+07	8.04E+06	5.72E+06	5.22E+07		
V	033-010	3.68E+07	9.10E+04	1.16E+05	1.29E+05	1.67E+05	7.83E+05
V	035-003	1.95E+08	5.24E+06	1.20E+07	2.01E+06	2.57E+07	2.21E+09
V	045-001	4.62E+06	5.45E+03	1.79E+04		6.96E+08	

V	002-004	4.10E+09	8.59E+05	3.34E+05	2.86E+08		7.33E+06
V	002-011	6.71E+07	3.37E+05	1.46E+05	2.61E+08	5.14E+08	
V	002-015	2.67E+08	1.10E+05	1.28E+05			3.37E+05
V	002-016	3.95E+06	6.03E+05	1.17E+05			
V	009-001						
V	009-003	4.08E+07	1.20E+07	1.01E+06	1.30E+09	1.77E+08	2.16E+08
V	009-004	3.50E+07	4.08E+04	3.82E+04	8.13E+07		
V	009-006	1.38E+09	2.46E+05	7.72E+04	5.97E+08	2.84E+07	
V	017-002	1.20E+08	1.69E+05	1.48E+05	5.58E+08	4.46E+09	
V	019-005	2.18E+08	1.12E+05	1.05E+05	7.37E+08	2.91E+08	
V	019-008	1.68E+09	2.34E+05	1.39E+06	2.13E+08	3.42E+07	

V	019-010		2.49E+06	1.00E+05	3.08E+07	3.16E+07	
V	019-013	2.00E+09	1.51E+05	5.30E+07	3.80E+08	7.28E+07	
V	019-014	3.43E+08	2.33E+05	3.47E+05	7.27E+06	1.40E+07	
V	021-003	3.76E+09		3.95E+05	1.19E+09		1.23E+09
V	030-004	4.34E+09	4.13E+06	1.13E+08		7.25E+08	
V	035-002	1.95E+05	7.99E+04	8.71E+04			1.82E+05
V	037-006		3.73E+06	7.36E+05	6.91E+05		
V	037-010	3.81E+09	2.14E+05	3.92E+08	3.63E+06		
V	037-016		2.71E+05	1.90E+05	4.49E+09	1.35E+09	6.72E+09
V	042-001	4.56E+08	1.49E+07	4.42E+05			
V	042-002	7.86E+07	2.08E+05	1.08E+05	2.14E+09		
	**Median**	**3.05E+08**	**2.40E+05**	**1.90E+05**	**3.33E+08**	**1.77E+08**	**1.12E+08**
	**Mean**	**1.26E+09**	**2.06E+06**	**2.68E+07**	**7.67E+08**	**7.00E+08**	**1.36E+09**
	**# of samples**	**18**	**20**	**21**	**16**	**11**	**6**

Prevotella

Study drug	Subject ID#	Baseline (day 1)	Day 5	End of treatment (day 10)	Day 25	End of study (day 40)	Sample taken at time of suspected recurrence (REC)
Ridinilazole (R)	002-013	1.44E+10	5.66E+09	6.31E+09	8.28E+09	8.07E+09	
R	007-002	2.05E+06	8.49E+03	1.86E+04	2.31E+05	1.26E+06	
R	009-002	2.58E+09	9.91E+06	2.54E+10	6.97E+08	7.75E+08	
R	017-003	1.80E+10	8.94E+09	1.59E+10		1.12E+10	
R	022-001		9.21E+09	1.08E+10	2.88E+09	2.77E+08	
R	031-002	1.99E+10		1.91E+10	7.81E+07		9.14E+07
R	033-008	8.96E+09	1.94E+07	2.72E+06	2.25E+06	2.14E+10	2.47E+06
R	033-011	6.45E+06	7.93E+06	2.31E+10	3.70E+10	2.43E+09	
R	038-001		2.58E+06	2.36E+09	1.47E+10	2.23E+10	
R	038-004	7.54E+06	7.66E+05	5.69E+05	6.67E+05		
R	042-003		8.19E+09	6.06E+09			
R	045-003	1.90E+06	6.66E+06	1.03E+07	6.71E+06	6.22E+06	

R	002-007	5.69E+08	2.03E+06	1.14E+10	2.82E+08	7.85E+08	
R	002-009	5.25E+09	4.01E+08	2.66E+08	3.04E+09	5.69E+09	
R	002-012	1.21E+10	2.42E+10	1.34E+10			
R	009-005	7.46E+05	1.27E+05	4.51E+05	1.48E+06	1.02E+10	
R	009-007	2.09E+07	7.28E+09	3.05E+09	8.37E+09	1.47E+09	
R	019-001	1.63E+09	4.28E+09	5.01E+09	8.57E+06	1.31E+07	
R	019-003	9.31E+08	3.39E+09	5.27E+09	2.85E+09	1.91E+09	1.25E+10
R	019-006	9.97E+08	1.46E+09	4.16E+09	4.51E+09	8.59E+09	
R	019-007	1.44E+10	7.71E+09	6.46E+09	1.62E+10	1.80E+10	
R	019-009	2.95E+09	6.17E+09	6.53E+09	1.15E+10	1.89E+10	
R	021-001	3.36E+09	1.63E+09	5.42E+09	4.21E+06	4.49E+09	2.18E+09
R	029-002	5.84E+09	3.55E+09	1.18E+09	8.23E+09	1.13E+09	6.41E+09
R	030-005	5.96E+09	1.57E+10	7.78E+09	3.15E+09	3.76E+08	
R	031-001	7.95E+09	9.12E+09	7.67E+09			
R	032-003	3.44E+09	1.17E+10	1.99E+06	4.37E+06	7.88E+07	

R	033-009	5.24E+07	2.14E+10	8.49E+09	3.77E+10	2.48E+10	
R	035-001	2.58E+05	1.55E+06	1.69E+06	2.52E+06	1.26E+10	
R	036-001	2.49E+06	2.16E+05	4.38E+06	3.79E+06	1.02E+10	
R	037-004	5.01E+09	1.29E+07	2.55E+07	7.83E+06	7.13E+06	
R	037-008	1.66E+06	1.81E+06	1.32E+06	1.18E+06		
R	037-014		8.21E+08	2.06E+08	4.20E+05	3.08E+09	
R	037-015	4.97E+10	1.44E+09	1.06E+10	2.04E+09	2.75E+09	
	**Median**	**2.95E+09**	**2.51E+09**	**4.59E+09**	**1.16E+09**	**3.08E+09**	**2.18E+09**
	**Mean**	**5.72E+09**	**5.47E+09**	**4.41E+09**	**4.89E+09**	**6.58E+09**	**7.05E+09**
	**# of samples**	**21**	**22**	**22**	**20**	**19**	**3**

V	002-014	2.82E+06	7.13E+06	5.41E+08			
V	007-001	2.51E+09	5.91E+05	3.86E+05	2.70E+09	4.41E+09	
V	009-010	1.59E+10	6.15E+07	1.92E+07	3.07E+07	1.21E+09	
V	019-004	3.50E+09	1.15E+07	6.34E+07			
V	029-001	3.87E+07	1.09E+05	5.36E+04	1.06E+06	4.64E+04	
V	029-004	1.97E+09	4.80E+05	1.45E+06	6.77E+09	1.37E+10	
V	033-007	2.97E+08	6.80E+09	3.48E+09	1.81E+09		
V	033-010	4.72E+09	1.55E+06	2.56E+06			6.41E+06
V	035-003	3.85E+09	4.50E+07	9.05E+07	5.35E+06	5.68E+08	1.95E+10
V	045-001	5.04E+08	8.90E+04	5.91E+04		4.40E+09	

V	002-004	6.23E+09	1.51E+06	5.25E+06	1.33E+06		1.57E+06
V	002-011	2.42E+08	5.41E+06	4.63E+05	3.25E+08	1.49E+09	
V	002-015	5.18E+09	5.63E+06	7.99E+06			1.34E+07
V	002-016	1.90E+08	2.22E+07	1.80E+07			
V	009-001	3.61E+06	3.40E+06	8.37E+06	3.65E+06	1.71E+07	
V	009-003	3.54E+08	2.59E+07	6.21E+06	2.33E+09	1.05E+09	4.78E+09
V	009-004	1.03E+09	2.77E+05	2.12E+05	1.71E+09		
V	009-006	2.10E+09	4.33E+05	1.21E+06	2.77E+06	6.06E+06	
V	017-002	6.56E+08	2.10E+07	6.99E+06	3.88E+09	1.73E+10	
V	019-005	3.81E+09	1.85E+06	1.11E+06	4.34E+09	5.41E+09	
V	019-008	9.31E+09	3.85E+06	6.25E+06	8.19E+08	1.30E+10	

V	019-010		3.16E+07	5.00E+05	1.22E+09	1.03E+10	
V	019-013	1.39E+10	2.69E+06	5.16E+09	8.29E+06	2.54E+10	
V	019-014	9.52E+08	1.07E+06	1.66E+06	1.52E+08	3.15E+08	
V	021-003	8.17E+09		1.06E+10	1.91E+10		1.97E+10
V	030-004	3.75E+10	2.19E+07	9.40E+06		2.56E+10	
V	035-002	2.88E+08	1.36E+08	1.41E+08			2.79E+06
V	037-006		1.89E+07	8.31E+06	3.12E+06		
V	037-010	8.27E+09	5.76E+09	6.28E+09	3.26E+07		
V	037-016		2.84E+06	3.43E+06	4.01E+09	2.41E+09	1.54E+10
V	042-001	1.16E+09	4.02E+07	2.64E+09			
V	042-002	4.28E+08	1.99E+07	5.12E+06	8.29E+09		
	**Median**	**1.16E+09**	**5.63E+06**	**6.62E+06**	**8.19E+08**	**3.91E+09**	**2.40E+09**
	**Mean**	**5.25E+09**	**2.92E+08**	**1.13E+09**	**2.72E+09**	**8.52E+09**	**6.66E+09**
	**# of samples**	**19**	**21**	**22**	**17**	**12**	**6**

Normal Stools

Sample	Bacteroides	Eubacteria	Enterobacteriaceae	*C. coccoides*	*C. leptum*	Prevotella
76A	1.31E+07	2.64E+09	2.06E+06	5.60E+09	1.92E+09	7.56E+06
30	4.60E+10	4.70E+10	4.75E+06	8.11E+09	4.68E+09	3.28E+10
6A	1.15E+10	1.32E+10	7.15E+06	2.50E+09	1.49E+09	7.70E+09
87A	7.97E+09	2.61E+10	3.78E+06	1.05E+10	2.96E+09	6.65E+09
66A	2.94E+10	2.44E+10	1.29E+08	5.99E+09	4.46E+09	1.67E+10
25	9.82E+09	1.50E+10	1.16E+08	3.32E+09	2.21E+09	6.25E+09
39	5.56E+10	6.39E+10	9.66E+06	2.52E+09	1.61E+09	4.82E+10
13A	2.42E+10	3.84E+10	7.85E+05	1.03E+10	4.86E+09	1.65E+10
105A	2.30E+10	2.99E+10	2.98E+06	5.90E+09	4.07E+09	2.38E+10
99A	5.07E+09	4.22E+09	1.33E+08	1.01E+09	1.31E+08	2.61E+09
134	8.14E+09	6.78E+09	4.67E+07	4.77E+09	2.44E+09	4.74E+09
137	7.32E+09	4.88E+07	1.28E+06	2.73E+08	8.10E+07	1.44E+09
140	6.55E+09	2.02E+10	9.52E+05	6.49E+09	4.38E+09	1.26E+10
143	2.29E+10	1.51E+10	2.25E+07	7.03E+09	1.26E+09	1.30E+10
**Median**	**1.06E+10**	**1.76E+10**	**5.95E+06**	**5.75E+09**	**2.33E+09**	**1.02E+10**
**Mean**	**1.84E+10**	**2.19E+10**	**3.43E+07**	**5.31E+09**	**2.61E+09**	**1.38E+10**
