# Supplementary material for: Enhanced preservation of the human intestinal microbiota by ridinilazole, a novel Clostridium difficile-targeting antibacterial, compared to vancomycin
Source: PLoS One. 2018 Aug 2;13(8):e0199810. doi: 10.1371/journal.pone.0199810 (PMC6071993; doi:10.1371/journal.pone.0199810)
Supplement: S5 Table — (DOCX) [file pone.0199810.s007.docx]

**S5 Table**

| **Coefficient** | **N** | **N.not.0** | **P-value** | **Q-value** | **Feature** |
| --- | --- | --- | --- | --- | --- |
| −0.01294 | 43 | 36 | 0.002 | 0.013 | **Actinobacteria** |
| −0.00785 | 43 | 21 | <0.0001 | <0.001 | c_Coriobacteriia f_Coriobacteriaceae |
| −0.00455 | 43 | 13 | 0.000 | 0.003 | *Eggerthella lenta* |
| −0.74493 | 43 | 42 | <0.0001 | <0.001 | **Bacteroidetes** |
| −0.64609 | 43 | 39 | <0.0001 | <0.001 | g_*Bacteroides* |
| −0.05547 | 43 | 20 | 0.001 | 0.008 | *Bacteroides ovatus* |
| −0.11691 | 43 | 21 | 0.001 | 0.010 | *Bacteroides uniformis* |
| −0.00888 | 43 | 16 | 0.003 | 0.019 | **Firmicutes** f_Clostridiaceae |
| −0.10853 | 43 | 31 | 0.001 | 0.007 | f_Lachnospiraceae |
| −0.05594 | 43 | 23 | 0.002 | 0.012 | g_*Blautia* |
| −0.00946 | 43 | 18 | 0.000 | 0.003 | *Blautia producta* |
| −0.01114 | 43 | 20 | 0.000 | 0.003 | g_*Coprococcus* |
| −0.00967 | 43 | 19 | 0.001 | 0.007 | g_*Dorea* |
| −0.03103 | 43 | 24 | <0.0001 | <0.001 | g_*Ruminococcus* |
| −0.03202 | 43 | 21 | <0.0001 | <0.001 | *Ruminococcus gnavus* |
| −0.06263 | 43 | 26 | 0.000 | 0.001 | f_Ruminococcaceae |
| −0.02985 | 43 | 21 | 0.001 | 0.005 | g_*Oscillospira* |
| −0.00981 | 43 | 16 | <0.0001 | <0.001 | g_*Ruminococcus* |
| −0.00553 | 43 | 13 | 0.001 | 0.005 | g_*Coprobacillus* |
| −0.01077 | 43 | 18 | 0.000 | 0.003 | g_*Eubacterium* |
| −0.01077 | 43 | 18 | 0.000 | 0.003 | *Eubacterium dolichum* |

Univariate analysis was performed in MaAsLin assessing both treatment groups at baseline and EOT. The coefficient indicates the direction of the association.

N= # of subjects; N.not.0=# of samples where value was not 0; q= p-value, corrected for false discovery rate
