# Supplementary material for: Enhanced preservation of the human intestinal microbiota by ridinilazole, a novel Clostridium difficile-targeting antibacterial, compared to vancomycin
Source: PLoS One. 2018 Aug 2;13(8):e0199810. doi: 10.1371/journal.pone.0199810 (PMC6071993; doi:10.1371/journal.pone.0199810)
Supplement: S6 Table — (DOCX) [file pone.0199810.s008.docx]

**S6 Table**

| **Coefficient** | **N** | **N.not.0** | **P-value** | **Q-value** | **Feature** |
| --- | --- | --- | --- | --- | --- |
| 0.003622 | 43 | 11 | <0.0001 | <0.001 | p**_Bacteroidetes** *Prevotella melaninogenica* |
| 0.01756 | 43 | 42 | 0.003 | 0.019 | p_**Firmicutes**  c_Bacilli |
| 0.017796 | 43 | 42 | 0.003 | 0.017 | o_Lactobacillales |
| 0.021118 | 43 | 27 | <0.0001 | <0.001 | f_Lactobacillaceae |
| 0.020542 | 43 | 27 | <0.0001 | <0.001 | g_*Lactobacillus* |
| 0.005931 | 43 | 12 | 0.002 | 0.014 | *Lactobacillus plantarum* |
| 0.015758 | 43 | 22 | 0.000 | 0.001 | *Lactobacillus zeae* |
| 0.010769 | 43 | 41 | 0.004 | 0.023 | f_Streptococcaceae |
| 0.140454 | 43 | 41 | 0.007 | 0.034 | f_Veillonellaceae |
| 0.1341 | 43 | 39 | 0.001 | 0.007 | f_Veillonellaceae g_*Veillonella* |
| 0.097058 | 43 | 39 | 0.001 | 0.007 | *Veillonella dispar* |
| 0.552907 | 43 | 43 | <0.0001 | <0.001 | **Proteobacteria** |
| 0.498926 | 43 | 43 | <0.0001 | 0.001 | c_Gammaproteobacteria |
| 0.556575 | 43 | 43 | <0.0001 | <0.001 | f_Enterobacteriaceae |
| 0.004736 | 43 | 21 | 0.004 | 0.023 | *Buttiauxella noackiae* |
| 0.068326 | 43 | 33 | 0.001 | 0.009 | g_*Citrobacter* |
| 0.009753 | 43 | 18 | 0.000 | 0.003 | *Citrobacter freundii* |
| 0.011586 | 43 | 20 | 0.005 | 0.026 | *Citrobacter youngae* |
| 0.027266 | 43 | 29 | 0.001 | 0.009 | g_*Enterobacter* |
| 0.020525 | 43 | 25 | 0.006 | 0.034 | *Enterobacter aerogenes* |
| 0.005464 | 43 | 16 | <0.0001 | <0.001 | *Enterobacter asburiae* |
| 0.009475 | 43 | 20 | 0.001 | 0.010 | *Enterobacter ludwigii* |
| 0.01252 | 43 | 26 | 0.002 | 0.013 | g_*Erwinia* |
| 0.194126 | 43 | 36 | <0.0001 | <0.001 | g_*Klebsiella* |
| 0.038105 | 43 | 26 | <0.0001 | <0.001 | *Klebsiella oxytoca* |
| 0.007055 | 43 | 16 | <0.0001 | <0.001 | g_*Pantoea* |
| 0.003012 | 43 | 7 | 0.004 | 0.023 | *Pantoea agglomerans* |
| 0.00486 | 43 | 12 | 0.002 | 0.013 | *Pantoea dispersa* |
| 0.01839 | 43 | 23 | <0.0001 | <0.001 | g_*Salmonella* |
| 0.018128 | 43 | 23 | <0.0001 | <0.001 | *Salmonella enterica* |
| 0.012729 | 43 | 24 | 0.006 | 0.030 | g_*Serratia* |
| 0.002353 | 43 | 6 | 0.008 | 0.041 | *Serratia ureilytica* |

Univariate analysis was performed in MaAsLin assessing both treatment groups at baseline and EOT. The coefficient indicates the direction of the association.

N= # of subjects; N.not.0=# of samples where value was not 0; q= p-value, corrected for false discovery rate
